# Boosting Fast‐Charging Performance of Ni‐Rich NCM9055 Cathodes with Nb_2_O_5_ Dual Functional Modification

**DOI:** 10.1002/advs.202522771

**Published:** 2026-03-12

**Authors:** Tian Rao, Zhaowen Bai, Jian Wang, Yang Ren, Qingsong Weng, Zhongzhu Liu, Maxim Avdeev, Robson Monteiro, Luanna Parreira, Xuejie Huang, Guohua Chen, Yongming Zhu

**Affiliations:** ^1^ School of Chemical Engineering and Chemistry Harbin Institute of Technology Harbin Heilongjiang China; ^2^ School of Energy and Environment City University of Hong Kong Kowloon Hong Kong SAR China; ^3^ Songshan Lake Material Laboratory Dongguan Guangdong China; ^4^ Institute of High Energy Physics Chinese Academy of Sciences 19B Yuquan Road Beijing China; ^5^ Department of Physics JC STEM Lab of Energy and Materials Physics City University of Hong Kong Hong Kong China; ^6^ Department of Mechanical Engineering, Research Institute for Smart Energy (RISE) The Hong Kong Polytechnic University Kowloon Hong Kong SAR China; ^7^ CITIC Metal Room 1901, Capital Mansion, Chaoyang Beijing China; ^8^ Australian Nuclear Science and Technology Organization (ANSTO) New Illawarra Road Lucas Heights New South Wales Australia; ^9^ CBMM North America Inc. Houston Texas USA; ^10^ Companhia Brasileira de Mineração e Metalurgia Araxã Brazil; ^11^ Department of Chemical and Biological Engineering The Hong Kong University of Science and Technology Clear Water Bay Kowloon Hong Kong SAR China

**Keywords:** fast‐charging, Li^+^ batteries, Nb_2_O_5_, Ni‐rich cathode materials, particle design, phase segregation

## Abstract

Polycrystalline Ni‐rich layered oxides are promising cathodes for Li‐ion batteries of high‐power density and long cycle life. However, their practical application is still hindered by the sluggish Li^+^ diffusion rate and reaction inhomogeneity during redox cycles. In this work, LiNi_0.9_Co_0.05_Mn_0.05_O_2_ (NCM9055) cathode with a desired internal radial structure was designed and successfully synthesized using Nb_2_O_5_ as a dual‐functional structural and interfacial modulator. During calcination, the Nb_2_O_5_ reacts to form an intergranular LiNbO_3_ phase at grain boundaries. This phase, forming before high‐temperature grain growth, acts as a structural modulator to preserve the desirable radial alignment of primary particles by impeding random grain growth. It also functions as an interfacial conductor, creating fast Li^+^ diffusion pathways along the grain boundaries. These structural and interfacial modifications synergistically mitigate chemical inhomogeneity and relieve accumulated strain during cycling. Consequently, the Nb‐modified NCM9055 exhibits superior electrochemical performance, delivering an excellent rate capacity (152.4 mA h g^−^^1^ at 10 C) and robust cycling stability under high‐rate conditions (83.0% capacity retention after 500 cycles at 5C). These findings clarify the mechanism of Nb modulation and demonstrate a robust strategy for preserving desirable microstructures in high‐rate, Ni‐rich cathode materials.

## Graphical abstract

A dual‐functional Nb_2_O_5_ modulation strategy is reported to overcome kinetic limitations in Ni‐rich cathodes. The formation of LiNbO_3_ phase at the grain boundary of the polycrystalline NCM9055 cathode preserves a radially aligned microstructure and establishes fast lithium‐ion pathways. These synergistic effects alleviate internal strain and mitigate chemical inhomogeneity, delivering an exceptional fast‐charging performance and a robust cycling stability for next‐generation batteries.

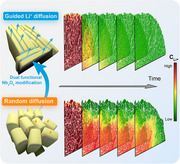

## 1 Introduction

Li‐ion batteries (LIBs) are widely applied to power portable electronics including the recent applications of electric vehicles (EVs) and drones [1]. However, the ever‐expanding market demand requires substantial advancements in battery technology, especially in high power density scenarios. For example, the range anxiety and considerably long charging time are challenges facing EV batteries [2, 3]. The extreme fast charging (XFC) technology that requires an 80% state of charge (SoC) in 15 min calls for innovative battery materials [4, 5, 6]. Nowadays, anode materials such as Titanium‐based and Niobium‐based ones like Li_4_Ti_5_O_12_ (LTO), TiNb_2_O_7_ (TNO) and LiNb_3_O_8_ (LNO) have demonstrated exceptional high‐rate performance with power densities exceeding 10 kW kg^−1^, thanks to their rapid lithium‐ion diffusion kinetics and structural stability during fast charge and discharge cycles [7, 8, 9]. In contrast, the advancement of compatible fast‐charging cathode materials is comparatively lagged behind. Ni‐rich cathode materials, such as LiNi_0.8_Co_0.1_Mn_0.1_O_2_ (NCM811) suffer from sluggish lithium‐ion kinetics and deteriorated performance when used under fast‐charging scenarios [10]. This disparity in fast‐charging capabilities between advanced anodes and current cathodes limits the overall performance of lithium‐ion batteries in applications requiring rapid energy delivery, underscoring the necessity for the development of fast‐charging cathode materials.

High Ni content in nickel‐rich layered cathode materials enhances their specific capacity due to the increased availability of Ni^2^^+^/Ni^4^^+^ redox couples. But this improvement comes with trade‐offs in their thermal stability, cycle life, and particularly fast‐charging capabilities, especially when Ni content is >90% [11]. The sluggish diffusion of Li^+^ is a key factor limiting the performance of high‐Ni content materials under fast‐charging conditions [12]. During rapid charging, limited Li^+^ mobility results in inhomogeneous charge and chemical distributions at different scales, accompanied by variations in the valence states of transition metals. This inhomogeneity in SoC and chemical states of transition metals can lead to localized overcharging and polarization, accelerating degradation of the cathode material [13]. Moreover, the repeated volumetric changes associated with fast Li^+^ insertion and extraction induce mechanical stress, causing cracks in the secondary particles as the rate of charging increases [14]. These cracks facilitate deeper electrolyte penetration, exacerbating a series of chained side reactions [15, 16, 17]. Structurally, fast charging increases local structural distortions around the NiO_6_ octahedra, which hinders Li^+^ diffusion pathways and undermines structural integrity during fast redox cycles [18].

Different strategies have been proposed to improve the diffusion of Li^+^ in Ni‐rich materials under fast‐charging conditions including surface engineering [19, 20, 21], structural modifications [22], and elemental doping [23]. For elemental doping, Nb has emerged as a particularly compelling dopant due to its reported ability to suppress volume changes and decrease particle size, as proved by both experimental and theoretical evidences [24, 25]. However, a critical knowledge gap remains regarding the interplay between Nb doping and surface coating formation. A clear analysis on the roles that Nb plays is needed to differentiate its incorporation into the bulk lattice and its segregation as a surface layer, such as LiNbO_3_. The precise fraction of Nb participating in each region, the underlying mechanisms governing its distribution, and the subsequent impact of LiNbO_3_ on the morphology remain largely unexplored [26].

An internal structure design of aligning primary particles inside polycrystalline spheres radially has been proposed and drawn much attention in research. Such a structure can alleviate the accumulated strain [27], decrease the tortuosity of Li^+^ transportation between primary particles [28] and suppress transition metal dissolution [29]. Several methods have been reported to create radially‐aligned primary internal particle structure including transition metal full concentration gradient (FCG) [30], tuning the concentration of ammonium during coprecipitation [31] and high valence ion doping [32]. Many of the methods introduce electrochemically inactive doping elements that partially decrease the reversible capacity of cathode. Report showed that at temperatures of >700°C the radial structure often fails to preserve after cathodes were calcined [33], although an initial radial alignment has been created through methods like those previously mentioned.

In this work, a novel LiNi_0.9_Co_0.05_Mn_0.05_O_2_ (NCM9055) material is presented with niobium oxide (Nb_2_O_5_) as a dual‐functional modulator to overcome the limitations of the sluggish Li^+^ diffusion and to enhance the electrochemical performance of NCM9055. The Nb modification successfully preserved the internal radial structure from precursors, which not only decreases the tortuosity of Li^+^ diffusion between particles by aligning diffusion pathways with the crystal planes but also relieves accumulated strain from the H2‐H3 phase change. To elucidate the underlying mechanisms, the modification process of Nb_2_O_5_ during calcination was investigated and the incorporation sites of Nb within the material were identified. High‐resolution synchrotron X‐ray diffraction (SXRD) and powder neutron diffraction (PND) analyses confirm that a significant fraction of Nb partially substitutes into the lattice, with the remaining forming LiNbO_3_ within the grain boundaries, serving as a fast ion conductor. As a result of these synergistic effects, the Nb‐modified material exhibits a significantly improved electrochemical performance, delivering a high capacity of 152.4 mAh g^−^^1^ at a 10 C rate, higher than the unmodified NCM9055 (139.8 mAh g^−1^). It also demonstrates an impressive capacity retention of 83.0% after cycling at 5 C for 500 cycles, compared to 59.8% for the unmodified NCM9055. The proposed mechanism underscores the potential of using Nb as a dual‐functional modulator, offering valuable insights for the future development of high‐performance Ni‐rich cathode materials, particularly in fast‐charging applications.

## 2 Results and Discussion

### 2.1 Radial Structure Preservation and LiNbO_3_ Phase Formation

**FIGURE 1:**
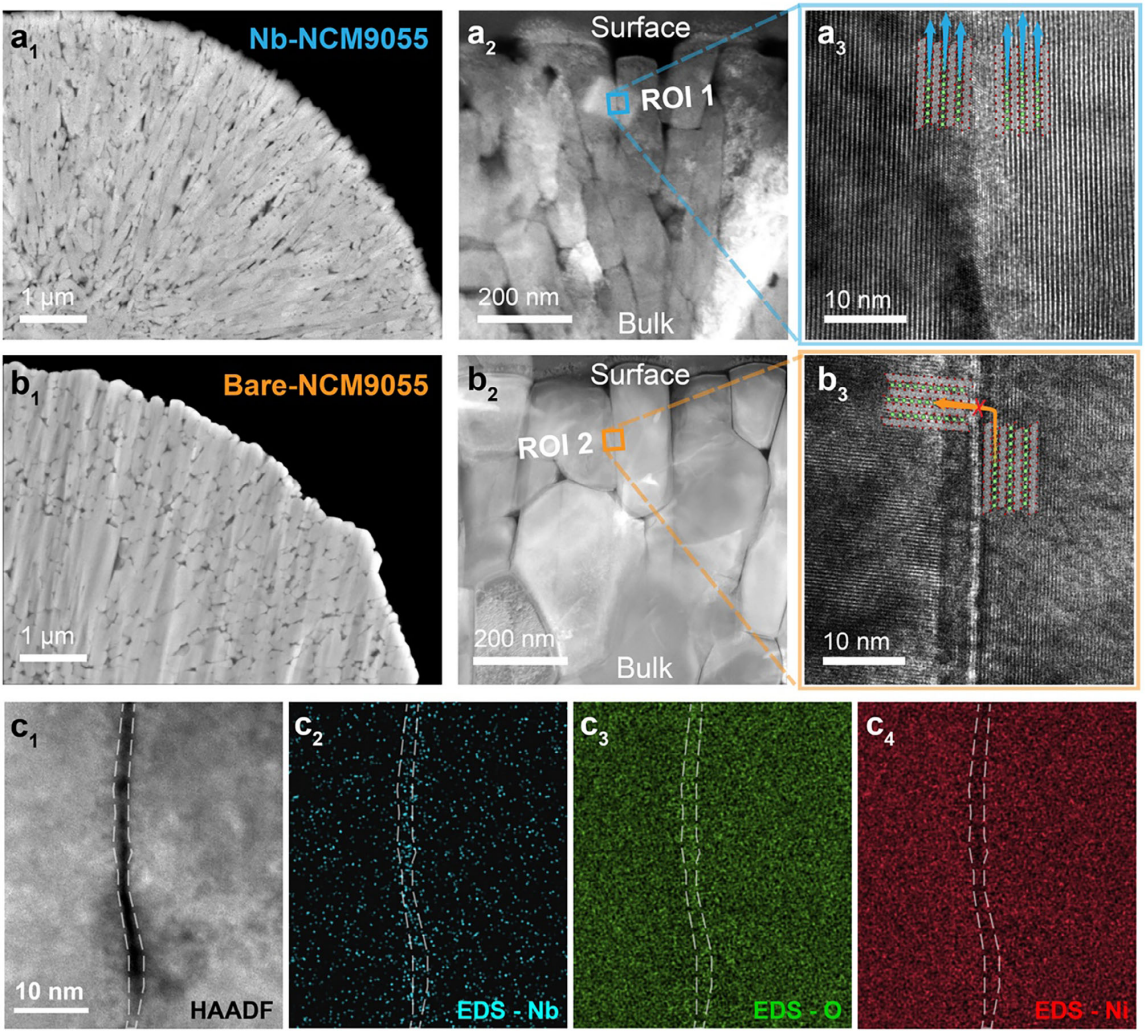
Cross‐sectional FESEM image of (a_1_) Nb‐NCM9055 and (b_1_) Bare‐NCM9055, respectively. STEM of particle cross‐section and HR‐TEM image on grain boundary of (a_2_,a_3_) Nb‐NCM9055; (b_2_,b_3_) Bare‐NCM9055 sample. (c_1_–c_4_) TEM‐HAADF image and EDS mapping at primary particle grain boundary of Nb‐NCM9055 sample. The grain boundary area is outlined by the white dashed lines.

The internal structure of Ni‐rich materials plays a crucial role in determining their electrochemical performance, particularly concerning the Li^+^ diffusion coefficient and cyclic stability [30]. In this study, radial alignment of primary particles was achieved via the co‐precipitation method by carefully adjusting the ammonia to NaOH ratio, as depicted by the scanning electron microscopy (SEM) images in Figures S1 and S2. This preferential alignment is likely influenced by the pristine ordering of the precursor on its (001) crystal plane, as free NH_3_ molecules adhere preferentially to the (001) plane during the co‐precipitation process, facilitating sheet formation in certain crystal direction [34, 35]. Precursors for NCM9055 cathode preparation were mixed with 0.5, 1, 3, and 5 mol% of Nb_2_O_5_ before calcination to test the effect of optimal Nb amount. After 4 h of pre‐calcination at 550°C and 15 h of high‐temperature calcination at 725°C under continuous O_2_ flow, the layered oxide structure was achieved. As shown in Figure S3, electrochemically, the 1 mol% set shows the most pronounced rate/cycling improvement, while 0.5 mol% gives only modest gains and ≥3 mol% begins to penalize capacity retention. The 1 mol% of Nb_2_O_5_ doping thus shows as the optimal modification amount. The chemical composition was validated by the inductively coupled plasma atomic emission spectroscopy (ICP‐AES) as listed in Table S1. The typical internal structure images of bare LiNi_0.9_Co_0.05_Mn_0.05_O_2_ (denoted as Bare‐NCM9055) and Nb modified Li[Ni_0.9_Co_0.05_Mn_0.05_]_0.99_Nb_0.01_O_2_ (denoted as Nb‐NCM9055) are available in Figures S4 and S5, respectively. Both samples display polycrystal spherical secondary particles with diameters of approximately 10 µm, composed of smaller rod‐shaped primary particles. After Nb modification, the size of primary particles of Nb‐NCM9055 is approximately 100 nm, much smaller than that of Bare‐NCM9055 sample (around 250 nm). Figures 1a1,b1 show the cross‐sectional field emission scanning electron microscopy (FESEM) images of Nb‐NCM9055 and Bare‐NCM9055, respectively. The Nb‐NCM9055 sample exhibits a distinct radial structure, characterized by elongated grains and well‐defined grain boundaries, in contrast to the unmodified sample. The voids observed between primary particles in the cross‐sectional images are intrinsic intergranular pores resulting from gas release and volume shrinkage during calcination. Quantitative analysis (Figure S6) was conducted on the cross‐sectional SEM image of primary particles shown in Figures 1a1,b1, which reveals a significant trend of elongation in length and shrinkage in width in the Nb‐NCM9055 sample, consistent with the particle surface morphology.

High‐resolution scanning transmission electron microscopy (STEM) and images near the grain boundaries between primary particles (Figures 1a2,a3,b2,b3) provide atomic‐level evidence that, the grains in the Nb‐modified NCM sample are preferentially aligned along the (003) crystal direction, which is the direction of Li^+^ diffusion [36]. This alignment facilitates guided diffusion channels from the bulk of the secondary particle to the cathode‐electrolyte interface at the surface. In contrast, the unmodified sample does not exhibit this preferential alignment, which might have been the result of isotropic crystal growth without the Li─Nb─O compound acting as impedance in grain boundaries [37]. As reported by Nomura et al. [38], Li^+^ transport relies on bulk diffusion through crystallographic planes like (003) rather than diffusion along grain boundaries. Hence, the observed preferential alignment along (003) in Nb‐NCM9055 sample avoids tortuous Li^+^ transport pathways in randomized crystal directions inside polycrystal particles, therefore enhances the efficiency of Li^+^ transportation for fast‐charging. To confirm the elemental distribution near the grain boundaries of primary particles, energy‐dispersive X‐ray spectroscopy (EDS) mapping was performed under transmission electron microscopy (TEM). An enrichment of Nb oxide near the grain boundaries is observed, as shown in high‐angle annular dark‐field (TEM‐HAADF) images in Figures 1c1–c4. To rigorously verify this distribution, STEM‐EDS line‐scanning was performed. The results displayed in Figure S7 reveal the distinct Nb signal peaks aligning with the grain boundaries, suggesting that Nb may not have totally incorporated into the lattice of the layered structure, consistent with previous reports [39, 40, 41].

**FIGURE 2:**
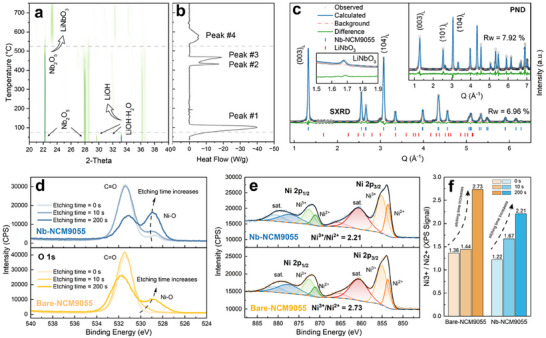
(a) In situ XRD pattern of Nb_2_O_5_ in LiOH·H_2_O from 25°C to 750°C and (b) corresponding DSC curve. (c) SXRD and PND patterns of Nb‐NCM9055 sample. The lower‐left inset is a magnified view of the SXRD pattern highlighting the LiNbO_3_ peak. The upper‐right inset is the PND pattern. (d) XPS O 1s spectrum depth profile of surface residual lithium compound layer of both samples. (e) XPS Ni ‐2p spectrum of both samples after 200 s etching and (f) Ni^3+^/Ni^2+^ ratio fitting results from depth profile with etching time of 0, 10, 200 s, respectively.

To clarify the configuration of the introduced Nb oxide species, in situ heating XRD was performed during the calcination process of the mixture of Nb_2_O_5_ and LiOH·H_2_O with a molar ratio of 1:2, under the same conditions of Nb‐NCM9055 preparation. By cross‐referencing with differential scanning calorimetry (DSC) results, four distinct phase shifts can be identified: dehydration of LiOH·H_2_O starting from approximately 76°C and peaking at 100°C (Peak #1), LiOH thermal decomposition at approximately 431°C (Peak #2), LiOH melting at approximately 465°C (Peak #3), and the formation of LiNbO_3_ starting from approximately 528°C, peaking at around 571°C and finishing at around 615°C (Peak #4). As illustrated in the in situ heating XRD diagram in Figure 2a and the enlarged view in Figure S8, the formation of a new material, LiNbO_3_, can be confirmed starting at approximately 525°C. The reactions between Li sources and Nb_2_O_5_ can be expressed as follows, with the reactant being LiOH or Li_2_O, depending on the temperature ramping rate:

(1) $$2\mathrm{LiOH}+{\mathrm{Nb}}_{2}O_{5}\;\to \;2\;{\mathrm{LiNbO}}_{3}+H_{2}O$$

(2) $${\mathrm{Li}}_{2}O+{\mathrm{Nb}}_{2}O_{5}\;\to \;2\;{\mathrm{LiNbO}}_{3}$$

To supplement the above characterizations, synchrotron XRD (SXRD) and powder neutron diffraction (PND) tests were conducted on the Nb‐NCM9055 cathode material, as shown in Figure 2c and its inset. A clear peak at Q = 1.679 Å^−1^ suggests that LiNbO_3_ is formed in the as‐prepared layered oxide samples during standard calcination. Such a peak was not found in Bare‐NCM9055 samples (Figure S9). Rietveld refinement was employed to quantify the exact amount of Nb within the lattice and in the form of LiNbO_3_, with results presented in Tables S2 and S3. It is observed that the lattice parameter *c* of Nb‐NCM9055 is slightly increased (14.20 Å, compared to the unmodified sample 14.19 Å), indicating that some Nb may have entered the lattice, substituting Ni. Detailed proportion analysis, cross‐checked with SXRD andPND results, reveals that approximately 89 atomic % of Nb substitutes Ni in the lattice, while the remaining 11 atomic % is present as LiNbO_3_.

To elucidate the role of Nb_2_O_5_ in the calcination process of Nb‐NCM9055 sample, the surface status of particles at different stages of calcination was examined using FESEM, as shown in Figure S10. Compared to bare samples (Figure S11), the primary particles are smaller from the onset of 725°C calcination (denoted as “0 hrs”, immediately following pre‐calcination, i.e., soon after the temperature reaches 725 ^°^C), indicating that the influence of the Nb modulator is already introduced at lower temperatures during pre‐calcination. Nano‐sized particles are evenly dispersed on the outer surface of the precursor material starting at 725°C calcination, which is not visible in bare samples. As the 725°C calcination continues, the amount of nano‐sized particles on surface decreases while a new gel‐like compound gradually appears. The final Nb‐NCM9055 material exhibits a more densely packed structure compared to the Bare‐NCM9055 material, with no other particles remaining on the surface. In addition, based on the lab XRD result shown in Figure S12, for 0.5, 1, 3 and 5 mol% introduction of Nb, with the increasing amount of Nb_2_O_5_ added, the signal of LiNbO_3_ increases as well.

**FIGURE 3:**
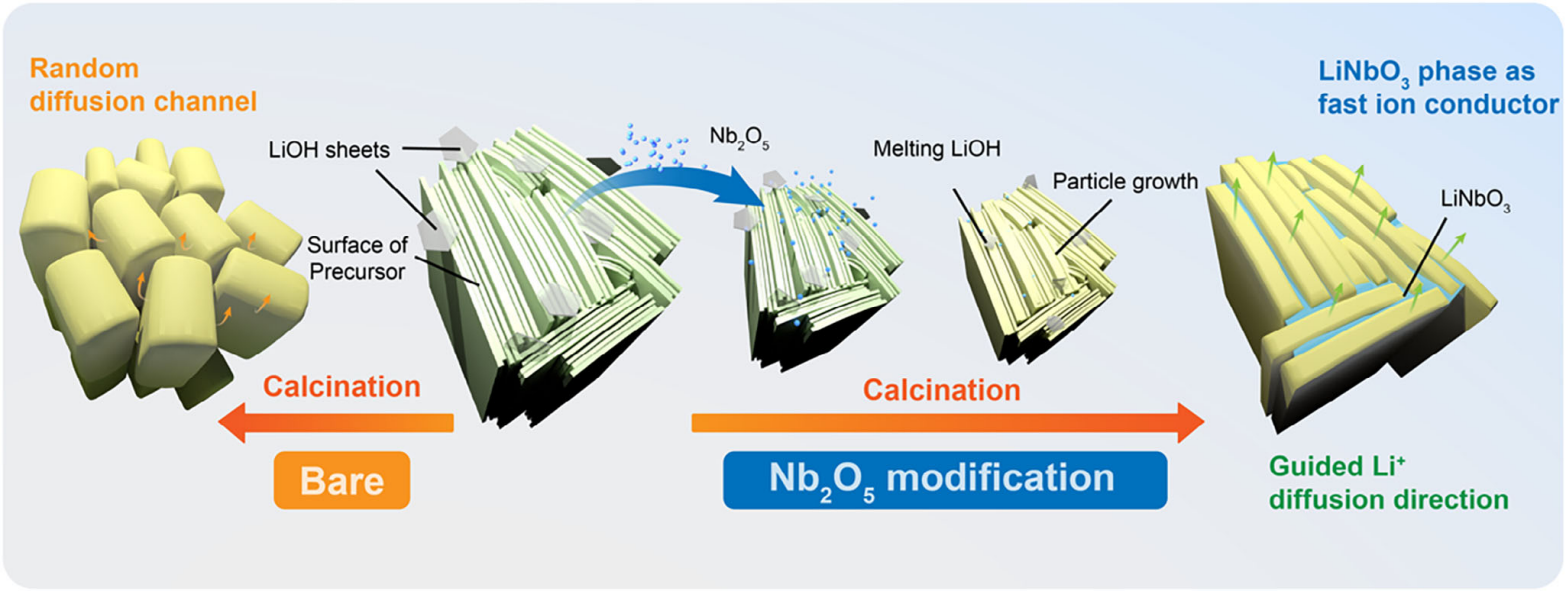
Schematic illustration of the morphology alternation mechanism with and without Nb_2_O_5_ modulator in calcination process.

X‐ray photoelectron spectroscopy (XPS) analyses were conducted on both Bare‐NCM9055 and Nb‐NCM9055 samples to investigate their surface properties. Previous studies have shown that surface residual lithium compounds (LiOH and Li_2_CO_3_), which are directly related to the Li / TM ratio during calcination, can adversely affect the initial coulombic efficiency and reversible capacity of cathode materials [42]. The O 1s peaks for both samples are found in Figure 2d. The Nb‐NCM9055 sample exhibits lower intensity for carbonated signals in both surface and sub‐surface depth profiles compared to the Bare‐NCM9055 samples, with enhanced performance expected as seen subsequently. The decreased carbonated compounds might be attributed to the formation of LiNbO_3_ that consumes more Li at surface [43]. Additionally, the XPS Ni 2p region for both materials are shown in Figure 2e. The Ni 2p_1/2_ and Ni 2p_3/2_ peaks can be interpreted as integrated peaks from Ni^2+^ and Ni^3+^ contents on the material. The fitted results for each depth profile, shown in Figure 2f, indicate that Nb‐NCM9055 possesses a thinner surface degradation layer composed of the rock‐salt NiO phase and residual lithium compounds, as the Ni^3+^/Ni^2+^ ratio is higher at 10 s etching but lower at 200 s etching, compared with Bare‐NCM9055. The elevated Ni^3+^/Ni^2+^ ratio at 10 s is consistent with the thinner surface degradation layer in Nb‐NCM9055, where the same etching depth already penetrated through to rxpose the underlying bulk layered phase that has higher Ni^3+^ content. The lower Ni^3+^/Ni^2+^ ratio observed at 200 s etching could be attributed to charge compensation resulting from the incorporation of high‐valence Nb^5+^, corroborating with the previous refinement data. Based on the analysis, the calcination process of Ni‐rich layered oxides can be summarized into four stages, as illustrated in Figure 3.

1. **Below 100°C**, LiOH·H_2_O undergoes dehydration and leaves behind LiOH.
2. **From approximately 431°C to 465°C**, the thermal decomposition of LiOH commences, while the residual LiOH melts at 462°C. The observed coexistence of thermal decomposition (Peak #2) and melting (Peak #3) of LiOH in Figure 2b can be attributed to the sluggish kinetics of its decomposition reaction, where residual LiOH persists beyond the decomposition temperature and subsequently undergoes melting [44].
3. **During pre‐calcination at 550°C**, a minor proportion of the introduced Nb_2_O_5_ on the surface begins to react with molten LiOH, or Li_2_O. This reaction leads to the formation of LiNbO_3_ at the grain boundaries and consumes additional Li sources. In this stage, the growth of the layered phase is limited due to the relatively low temperature, permitting the penetration of LiNbO_3_ into the grain boundaries.
4. **After pre‐calcination**, the calcination temperature is increased to 725°C and maintained for 15 h, during which the layered oxide phase undergoes significant crystal growth along the (003) plane under the influence of LiNbO_3_. Additionally, the residual Nb enters the lattice of the layered oxides.

To clarify the mechanism of the radial structure preservation, it is important to consider first the failure mode of the Bare‐NCM9055 sample. At the high calcination temperature of 725°C, the system is thermodynamically driven to minimize total surface energy. In the bare sample, this manifests as rapid isotropic grain growth and re‐orientation. The primary particles are free to coarsen, consuming smaller particles nearby and randomizing their alignment. This process, driven by atomic diffusion across the grain boundaries, is what destroys the radial structure of the precursor, resulting in the random particle alignment observed.

The success of our Nb‐NCM9055 sample hinges on the timing of the LiNbO_3_ formation. As the in situ heating XRD (Figure 2a) shows, it forms at ∼525°C, significantly before the 725°C stage where aggressive grain growth begins. This LiNbO_3_, confirmed existence on the grain boundaries by TEM‐EDS results (Figures 1c1–c4), acts as a thermodynamically stable physical barrier network. The stable, intergranular LiNbO_3_ particles physically impede the motion of the NCM grain boundaries. By doing so, they inhibit the atomic diffusion between grains so that the radial structure is preserved.

### 2.2 Electrochemical Performance and Fast‐Charging Capability

**FIGURE 4:**
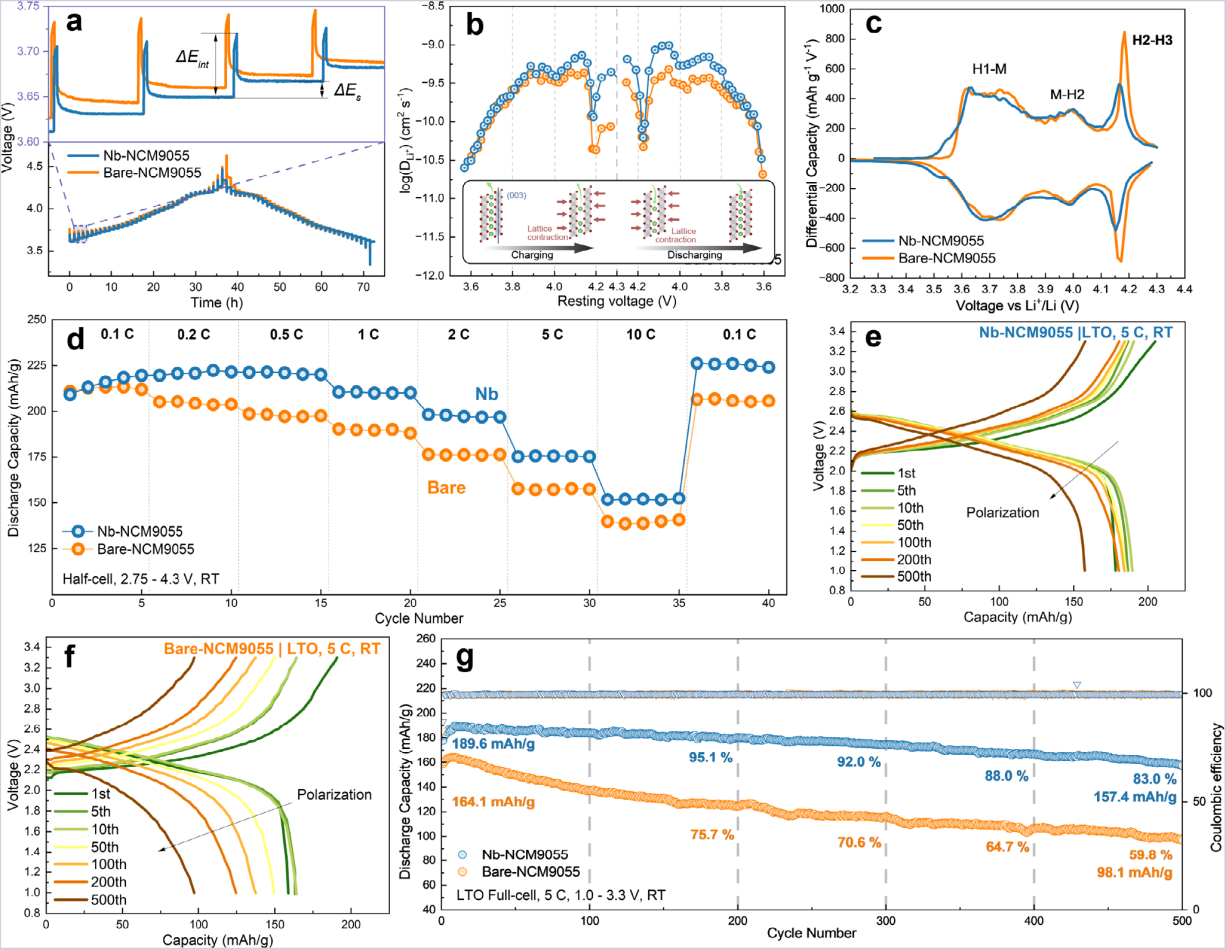
(a) The voltage response curve from GITT tests of Nb‐NCM9055 and Bare‐NCM9055 samples. (b) Calculated Li^+^ diffusion coefficient of Nb‐NCM9055 and Bare‐NCM9055 samples. (c) Differential capacity (dQ/dV) plot of both samples for the second cycle at 0.1 C using half‐cells. (d) Reversible discharge capacity of both samples at different rates. Voltage profiles showing polarization increases during redox cycle of (e) Nb‐NCM9055 sample and (f) Bare‐NCM9055 sample. (g) Cycling capacity retention and coulombic efficiency of both samples of LTO|NCM9055 full‐cells at 5 C.

To determine if the preserved radial structure translates to enhanced kinetics, the electrochemical performance of both Nb‐modified and bare samples was evaluated using half‐cell (with a lithium metal pellet as the anode) and full‐cell (with lithium titanium oxide, hereafter referred to as LTO, as the anode) configurations. To quantify the enhancements in Li^+^ diffusion resulting from Nb modification at various states of charge, the diffusion coefficients for Li^+^ in both samples were measured using the Galvanostatic Intermittent Titration Technique (GITT) on half‐cells. Intermittent electrochemical pulses were applied to recording the intermittent voltage (*ΔE_int_*) and steady‐state voltage (*ΔE_s_*) responses of the cells, as depicted in Figure 4a. The resulting data were calculated and plotted against the cell voltage. The Li^+^ diffusion in Ni‐rich layered cathodes proceeds through three stages: atomic movement via oxygen dumbbell hopping (ODH) or tetrahedral site hopping (TSH); transport through vacancy channels in primary particles; migration to secondary particle surfaces. As shown in Figure 4b, the calculated Li^+^ diffusion coefficients (D_Li+_) for both samples exhibit similar fluctuation trends during the charge and discharge cycles. The Nb‐NCM9055 sample demonstrates higher Li^+^ diffusion coefficients across most voltage regions, indicating a consistent improvement in Li^+^ transport efficiency. The Li^+^ diffusion coefficient measured at the start of charging and at the end of discharge for both samples are approximately 3.16 × 10^−11^ cm^2^ s^−1^, which might correspond to the slow Li^+^ and $V_{Li^{+}}^{\cdot }$ reordering process when the layer is nearly saturated with Li^+^ or $V_{Li^{+}}^{\cdot }$, consistent with previous reports [45, 46]. The improved Li^+^ kinetics in Nb‐modified samples can be attributed to enhanced transport through the radial structure, which aligns with the structural and morphological evidence presented above. In addition, the formed LiNbO_3_ at grain boundary might have a beneficial role in improving Li^+^ transportation [47], acting as a fast ion conductor layer inside the grain boundary. The observed expansion of the lattice parameter *c* might also contribute to this enhanced kinetic performance [48]. To quantitatively analyze the kinetics and identify the origin of the improved rate performance, electrochemical impedance spectroscopy (EIS) analysis was conducted. The Nyquist plots (Figure S13) were first fitted using an equivalent circuit model (Figure S14), and the quantitative results (Table S4) indicate that both the surface film resistance (R_sf_) and charge‐transfer resistance (R_ct_) are lower for the modified sample. To explicitly decouple these overlapping electrochemical processes without the ambiguity of circuit modeling, distribution of relaxation times (DRT) analysis was further performed (Figure S15). The DRT profile reveals distinct time constants corresponding to different kinetic steps. The dominant peak located in the frequency range of 10^−4^ to 10^−3^ s corresponds to the Li^+^ migration through the cathode electrolyte interphase (CEI). As observed, the Nb‐NCM9055 sample exhibits a significantly suppressed peak intensity in this region compared to the bare sample, confirming that the LiNbO_3_ coating effectively decreases interfacial resistance. Furthermore, the peaks in the medium frequency range (0.01 to 0.5 s), corresponding to the charge‐transfer process, also demonstrate a decreased intensity for the modified sample [49]. This consistent decrease in resistance across both the interface and charge‐transfer regions, corroborated with both the equivalent circuit fitting and DRT analysis, confirms that the Nb modification decreases the kinetic energy barriers, therefore enabling the superior fast‐charging capability. The differential capacity plots in Figure 4c reveal three distinct charging and discharging peaks from the second cycle onward for both Bare‐NCM9055 and Nb‐NCM9055 samples. These peaks correspond to the H1–M, M–H2, and H2–H3 phase transitions inherent in nickel‐rich layered oxide cathode materials. The H2–H3 peaks are significantly lower for the Nb‐NCM9055 samples, as the intensity of these peaks is often associated with the extent of volume change, which is a known factor contributing to microcracks and cycling‐induced degradation in Ni‐rich cathode materials [50]. Additionally, the discharge peak at approximately 3.5 V, which is present only in the Nb‐NCM9055 sample, is attributed to Li^+^ reordering with vacancies [51]. This suggests a higher degree of crystal stability, facilitating faster Li^+^ intercalation during the end of discharge. Consequently, this leads to a higher initial coulombic efficiency and an improved reversibility during subsequent cycling.

Figure 4d illustrates the reversible capacities at different cycling rates. The Nb‐NCM9055 sample exhibits superior capacities of 219.7 mAh g^−1^ at 0.1 C and 152.4 mAh g^−1^ at 10 C compared to the bare sample, which is top among reported Nb modified Ni‐rich cathode samples (Table S5). The change of the valences of Co and Mn elements has been checked with XPS spectrum in Figures S16–S19. No significant valence change of Co or Mn is observed in both Bare‐NCM9055 and Nb‐NCM9055 samples before and after charging, proving that nearly all the capacity was contributed by Ni redox process. Notably, the capacity difference between the Nb‐modified and bare samples is most significant at 2 C, with the Nb‐NCM9055 showing a significant capacity increase ratio. This disparity, evident at 2 C but not much at 10 C, may be attributed to the ionic conductivity of the carbonate‐based electrolyte and charge transfer resistance of lithium counter electrode becoming dominant limiting factors at extremely high rates such as 10 C, overshadowing the improvements made in the cathode material [52]. To ensure that the superior rate performance observed in half‐cells is intrinsic to the cathode and translates to practical cell configurations, rate capability tests were also conducted in LTO|NCM9055 full‐cells. As presented in Figure S20, the Nb‐modified full‐cell exhibits excellent discharge capacities of 170.5 and 154.1 mAh g^−1^ at 5 and 10 C, respectively. This robust rate performance in the full‐cell system confirms that the improved kinetics are driven by the optimized Nb‐NCM9055 cathode rather than artifacts of the lithium metal counter electrode. After high‐rate cycling, the Nb‐NCM9055 sample demonstrates a higher capacity recovery when the discharge rate returns to 0.1 C compared to its original 0.1 C capacity, indicating a greater resilience to the stresses induced by high‐rate operation. This suggests that Nb modification enhances structural reversibility and mitigates degradation mechanisms that typically impair performance under aggressive cycling conditions.

To further validate the high‐rate performance of the Nb‐NCM9055 cathode material, full‐cell cycling tests were conducted using LTO as the anode instead of the conventional graphite‐based material. The full‐cells were cycled at 5 C (1 C = 180 mA g^−1^), Figure 4g. Both materials present their highest discharge capacity after a few cycles of activation, which might be caused by the formation of cathode‐electrolyte interfacial layer [53]. After 500 cycles at a high rate of 5 C, the Nb‐NCM9055 electrode exhibits a remarkable capacity retention of 83.0%, corresponding to 157.4  mAh g^−1^, significantly outperforming the Bare‐NCM9055, which retains only 59.8% of its capacity at 98.1 mAh g^−1^. This substantial improvement demonstrates the excellent cycling stability of Nb‐NCM9055 under high current conditions, highlighting the effectiveness of Nb modification in enhancing structural integrity and electrochemical performance during prolonged high‐rate cycling. Extended cycling tests were also performed in half‐cell configurations, as shown in Figures S21 and S22, the Nb‐NCM9055 sample demonstrates exceptional stability compared to Bare samples at 1 C, retaining a high capacity of 198.3 mAh g^−1^ after 150 cycles with a retention rate of 94.6%. At the aggressive rate of 5 C, while the capacity decayed to 129.5 mAh g^−1^ after 300 cycles likely due to the degradation of the Li metal anode, it remained significantly higher than the Bare sample, confirming the improved stability of the cathode material itself. To further understand the effect of accumulated strain on cycling performance, ex situ XRD tests were conducted on electrodes of both samples before and after 500 cycles under 5 C, as shown in Figures S23 and S24. Both samples present with irreversible peak shift to lower angles, especially in (003) peak, which indicates the irreversible structural degradation on c‐axis direction. This could be resulted from the repetitive lattice expansion / contraction, causing the collapse of the overall structure and electrolyte penetration through microcracks. The Nb‐NCM9055 sample again presents with a much smaller irreversible change in terms of structural degradation, indicating its enhanced stability for high‐rate cycling. The polarization plots in Figures 4e,f indicate a much faster increase in polarization for the Bare‐NCM9055 compared to the Nb‐NCM9055 sample. The slower polarization observed in the Nb‐modified sample suggests its decreased internal resistance and improved lithium‐ion diffusion kinetics within the electrode during cycling. The increased polarization is often associated with sluggish ion transport and accumulation of impedance at electrode interfaces, which can accelerate capacity degradation [54]. Therefore, the mitigated polarization growth in Nb‐NCM9055 implies enhanced electrochemical stability and sustained high‐rate performance.

### 2.3 Mechanism of Homogenized Kinetics and Strain Alleviation

**FIGURE 5:**
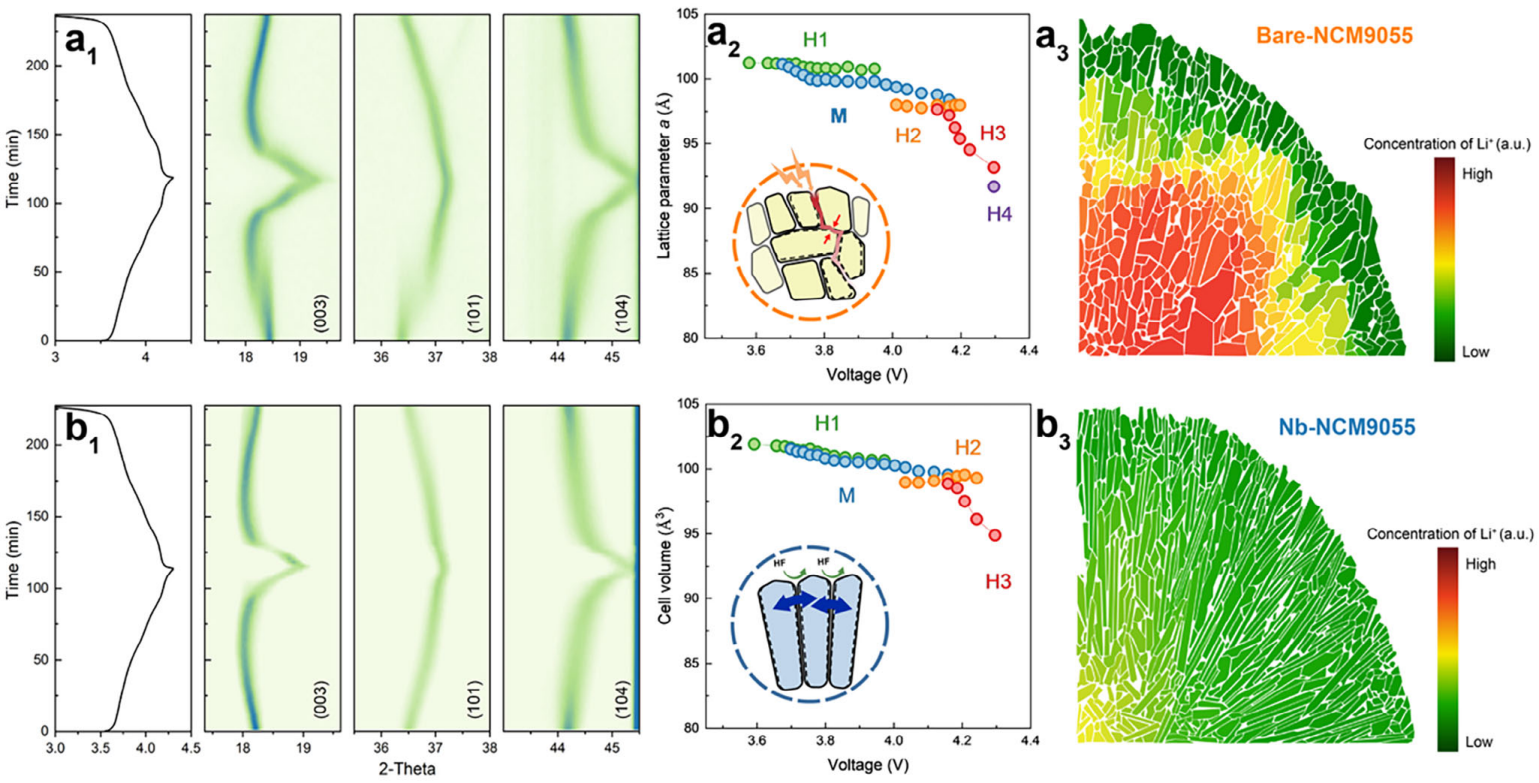
In situ XRD curve showing peaks shifting of (a_1_) Bare‐NCM9055 and (b_1_) Nb‐NCM9055 sample. The change on cell volume during charging of (a_2_) Bare‐NCM9055 and (b_2_) Nb‐NCM9055 samples from Rietveld refinement. The insets are schematic illustrations of the mechanism of volume change causing degradation. The Li^+^ diffusion simulation result of (a_3_) Bare‐NCM9055 with random particle alignment and (b_3_) Nb‐NCM9055 with radial structure.

The superior electrochemical performance observed above stems from fundamental changes in how the material accommodates Li^+^ diffusion in particles. To elucidate the underlying mechanism, in situ electrochemical XRD, post‐cycling SEM and diffusion simulations were utilized to link the performance improvements directly to the mitigation of chemical inhomogeneity and alleviation of the accumulated mechanical strain during charge–discharge cycles by the dual‐functional Nb_2_O_5_ modification. In situ electrochemical XRD was employed to study the effect of Nb on the cycling performance of Bare‐NCM9055 and Nb‐NCM9055 cathodes. Figures 5a1,b1 show distinct peak shifting behaviors. Nb‐NCM9055 exhibited smoother peak shifting than Bare‐NCM9055. Conversely, Bare‐NCM9055 showed phase segregation (separate peaks for different lattice parameters) throughout charging. The smoother peak progression in Nb‐NCM9055 suggests more uniform lattice parameters during delithiation, indicating enhanced structural integrity and more homogeneous Li^+^ diffusion pathways. Such phase segregation in Ni‐rich cathodes often implies uneven Li^+^ distribution or SoC inhomogeneities [55]. Suppressed phase segregation during XFC supports fast Li diffusion kinetics and high rate capability of Ni‐rich layered oxides [48]. Rietveld refinement of in situ XRD data (Figures 5a2,b2; S25 and S26) further details cell volume and lattice parameter changes. While overall cell volume changes during delithiation are similar (Bare‐NCM9055: 9.42%; Nb‐NCM9055: 6.89%), cell volume and lattice parameter *c* trends show a more gradual change in Nb‐NCM9055 during charging compared to Bare‐NCM9055. Furthermore, a near fully delithiated H4 phase, observed only in Bare‐NCM9055, reveals the evidence of the extreme chemical inhomogeneity in the unmodified sample. This improved phase stability in NCM9055 is primarily attributed to two factors: (1) The radial structure may facilitate more efficient Li‐ion diffusion, reduce diffusion polarization and yield a more consistent charging profile. Enhanced Li^+^ mobility mitigates local concentration gradients and alleviates internal stresses from phase segregation with differing lattice parameters. (2) Incorporated Nb reportedly decreases volume changes during Li^+^ intercalation/deintercalation [24]. This implies less lattice strain and fewer detrimental phase transitions, crucial for structural integrity and extended cycling life in high‐energy cathodes.

To assess the impact of different internal structures on fast‐charging capabilities, diffusion simulations were performed. The diffusion behaviors in the two distinct structures are shown in Figure 5a3,b3 and supplementary video clips. The radial structure of Nb‐NCM9055 sample exhibits more uniform and rapid Li^+^ diffusion, as indicated by fewer residual Li^+^ ions remaining within the particles in any given diffusion time. In contrast, the randomly distributed particle structure of the Bare‐NCM9055 sample exhibits significant concentration inhomogeneities due to the tortuosity of the diffusion pathways. This simulation result is experimentally verified by the in situ XRD patterns as detailed in Figure S27. The distinct peak splitting phenomenon observed only in the Bare‐NCM9055 sample indicates the detrimental coexistence of multiple phases (H1, M, H2, H3, and H4) each with a different lattice parameter at most of the apparent voltage range, especially around 3.80 V. This peak segregation serves as a direct fingerprint of chemical inhomogeneity—where different regions within a secondary particle possess different SoC although at the same macroscopic voltage. Such kinetic disparity not only induces local polarization but also generates significant lattice mismatch stress between coexisting phases, directly driving the accumulated strain and intergranular cracking responsible for performance degradation [55]. Conversely, the radial structure minimizes these gradients, ensuring a homogeneous solid‐solution reaction, and the straight and direct pathways from the particle core to the surface shortens diffusion path lengths and decreases tortuosity. Consequently, Li^+^ transport is accelerated, and the SoC within the particle becomes more homogeneous. As a result, Li^+^ concentration gradients are decreased, thereby decreasing internal resistance and preventing localized overpotentials and performance degradation. To validate the effect of alleviation of local inhomogeneity brought by Nb modification, SEM images of the electrodes after 300 cycles were examined (Figure S28). The Bare‐NCM9055 particles exhibit severe intergranular cracking and fragmentation. Conversely, the Nb‐NCM9055 particles remain mechanically intact, confirming that the suppression of phase inhomogeneity successfully mitigates the internal stress accumulation so that particle disintegration is decreased. Collectively, these findings highlight that managing volume change is critical for mitigating internal strain in cathode particles. Preventing microcrack initiation and propagation limits fresh surface exposure to the electrolyte. This, in turn, decreases the risk of detrimental side reactions like HF attack that penetrate particles, leach transition metals, and worsen structural degradation via cascading reactions [56, 57]. Suppressing crack formation allows Nb‐NCM9055 to mitigate these detrimental reactions and associated structural degradation, enhancing its overall structural integrity and safety. This confirms that Nb modification enhances mechanical robustness by suppressing crack initiation and growth, improves capacity retention and high‐rate cycling stability.

The mitigation of chemical inhomogeneity by the Nb modification is a synergistic effect among three key pieces of evidence. First, the successful presevation of the radial strcture as shown by the morphological images (Figures 1a1 and 1b1), providing aligned and direct pathways for Li^+^. Second, the diffusion simulation results (Figures 5a3 and 5b3) predict that the aligned structure would lead to a highly uniform reaction with minimized local polarization. Third, the in situ electrochemical XRD (Figures 5a1 and 5b1) experimentally confirms this homogeneity, showing smooth, continuous peak shifting and suppressed phase segregation. In contrast, the Bare‐NCM9055 sample loses its radial structure (Figure 1b1), resulting in a random and tortuous diffusion network that causes severe phase segragation and simultaneous state‐of‐charge variations. Ultimately, this electrochemical homogeneity in Nb‐NCM9055 prevents the localized over‐strain that leads to particle cracking, which explains the superior structural integrity of the Nb‐NCM9055 sample after cycling (Figure S22).

In summary, the Nb modification can significantly enhance electrochemical performance, structural stability, and mechanical integrity of NCM9055 cathode materials, mainly through two distinct mechanisms: (1) modulation of primary particle growth and preservation of the radial internal structure; (2) mitigation of chemical inhomogeneity by providing Li^+^ conducting LiNbO_3_ and decreasing Li^+^ diffusion tortuosity. These modifications contribute to decreased polarization, mitigated particle cracking, improved capacity retention, and sustained high‐rate performance. Collectively, these findings highlight Nb modification as a promising strategy for optimizing the rate performance and service life of Ni‐rich layered oxide cathodes in lithium‐ion batteries.

## 3 Conclusion

Nb modification of NCM9055 significantly enhances the fast‐charging performance and cycling stability of Ni‐rich cathode materials. The mechanism of such improvement could be attributed to two major ways: The LiNbO_3_ formation occurs at a temperature as low as approximately 525°C during calcination and plays a critical role in impeding the isotropic growth of primary particles, thereby preserving the radial structure of the precursor, although the majority of the introduced Nb element would substitute into the layered lattice. The successful preservation of the internal radial structure would facilitate rapid Li^+^ diffusion by decreasing the tortuosity of Li^+^ diffusion, relieve otherwise accumulated strain from anisotropic volume change during redox cycling and decrease the inhomogeneity inside particle during fast charging. Consequently, the Nb‐NCM9055 with 1 mol% Nb exhibits superior rate performance (189.6 mAh g^−1^ at 5C) and excellent capacity retention (83.0% after 500 cycles at 5C) compared to Bare‐NCM9055, demonstrating the effectiveness of Nb as a dual‐functional modulator for high‐performance Ni‐rich cathodes.

## 4 Methodology

### 4.1 Material Synthesis

The NCM9055 precursor with radially aligned structure was synthesized through a facile co‐precipitation method. A mixture of nickel sulfate, cobalt sulfate and manganese sulfate solution (2 mol/L), along with ammonia solution (3 mol/L) and sodium hydroxide solution (4 mol/L) were fed into an automatically controlled co‐precipitation device under continuous stirring. The change of ammonia concentration is controlled by regulating its feeding rate. The molar ratio of mixed transition metal solution, ammonia solution and sodium hydroxide was incrementally adjusted from 1:1.5:2 at the beginning of feeding to 1:3:2 at the end of feeding at a fixed increase rate. The pH was fixed to 11.50 using a customized automatic pH controller by adjusting the feeding rate of sodium hydroxide solution. The temperature was fixed to 50°C during the whole reaction process. After 36 h of reaction the feeding of all solutions was stopped, and the mixture was stirred for another 12 h to complete the reaction. The precipitate was recovered by vacuum filtration and dried in a convection oven for over 8 h at 60°C to remove the moisture.

The precursor powder was then blended with 105% stoichiometric amount of lithium hydroxide hydrate to compensate for the Li loss during grinding and calcination. The mixed powder was calcined in a tube furnace with continuous oxygen flow, first was heated up to 550°C for pre‐calcination for 4 h, before raising temperature to 725°C for 15 h. The heating and cooling rates were set to 5°C min^−1^. To synthesize Nb‐NCM9055, a desired molar ratio  of Nb_2_O_5_ was ground and mixed with LiOH⋅H_2_O before calcination.

### 4.2 Materials Characterization

Various advanced characterization techniques were employed to analyze the structural and morphological properties of the materials. The high energy X‐ray diffraction experiments were conducted at beamline P21.1, Deutsches Elektronen‐Synchrotron DESY. The wavelength of the high‐resolution X‐ray is 0.1223 Å, and the 2D diffraction patterns are obtained by a PerkinElmer large‐area detector in the transmission geometry. The distance between the sample and the detector is 1600 mm, and the 2D diffraction patterns were calibrated by standard CeO_2_. The 2D diffraction patterns are converted to 1D patterns by GSAS‐II. Powder neutron  diffraction (PND) was performed on the Echidna high‐resolution powder diffractometer at the Australian Nuclear Science and Technology Organization (ANSTO). Data for each sample was collected for 3 h at room temperature using a neutron wavelength of 1.6215 Å. For electrochemical in situ XRD, the lattice parameters and unit cell volumes of the pristine and modified samples were derived from the in situ XRD patterns via sequential Rietveld refinement. All Rietveld refinement procedures were performed using the GSAS‐II software package [58], the background was fitted using a Chebyshev polynomial function. The lattice parameters *c* and *a* were refined iteratively until the weighted R‐factor (R_w_) converged to a minimum value.

In situ heating XRD was performed using a customized setup on Bruker D8‐DISCOVER. The sample was first ground and mixed before mounting in position. The heating chamber was filled with air, and the temperature ramping rate was set to 5°C min^−1^. In situ XRD was performed using a PANalytical X'Pert^3^ lab diffractometer equipped with a customized in situ cell, enabling real‐time monitoring of structural changes during battery operation. TEM and STEM samples were prepared by focused ion beam (FIB) techniques, ensuring precise cross‐sectional imaging of the electrodes. Cross‐section SEM samples were prepared using argon‐ion cross‐polishing (Ar‐CP) to achieve high‐quality surface finishes.

For surface chemical analysis, XPS was conducted on pelletized powder samples, which were prepared by compression to achieve uniform and smooth surfaces. Additionally, Ar‐ion etching was used to examine depth profile of samples.

### 4.3 Electrochemical Performance Testing

For the fabrication of the cathode sheets, a specific mass ratio of 90:4.6:0.4:5 was used, corresponding to the active material, Super‐P conductive carbon, single‐wall carbon nanotube (SWCNT), and polyvinylidene fluoride (PVDF) binder, respectively. N‐methyl‐2‐pyrrolidone (NMP) served as the solvent for creating a homogeneous slurry. The resulting slurry was uniformly coated onto carbon‐coated aluminum foil and dried in a convection oven for 4 hrs at 60^°^C. The dried electrode was calendered to ensure proper adhesion and thickness. All procedures were conducted under controlled conditions in a dry room where the dew point was controlled to less than −30°C to minimize moisture interference. The prepared cathode sheets were punched with a model cutter with a diameter of 12 mm, achieving a mass loading of approximately 2 mg cm^−2^. The anode sheet was prepared similarly, with the active material replaced by lithium titanium oxide (LTO), using copper foil as the current collector.

For electrochemical testing, both half‐cells and full cells were assembled. The half‐cells are of a CR2032 configuration, with lithium metal as the anode, and Celgard 2325 as separator. The electrolyte solution was 1 m LiPF_6_ in a solvent mixture of diethyl carbonate (DEC), ethylene carbonate (EC) and dimethyl carbonate (DMC) in a 1:1:1 volume ratio, with 2 wt.% vinylene carbonate (VC) and 10 wt.% fluoroethylene carbonate (FEC) as additives. For full cells, the whole configuration is the same as half‐cells except substituting the lithium metal anode with an LTO anode. The capacity ratio of the negative and positive electrode (N/P ratio) is designed to be 1:1.2.

The electrochemical performance of the assembled cells was evaluated using a Neware battery testing system. Cycling, rate capability, and galvanostatic intermittent titration technique (GITT) tests were performed to analyze the performance of cells under different conditions. The current rate for the tests was standardized, with 1 C set to 180 mA g^−1^.

### 4.4 Diffusion Simulation

The diffusion model of bare‐NCM9055 and Nb‐NCM9055 samples was derived from the cross‐sectional SEM images presented in Figures 1a1,b1. Both samples were assumed to be fully lithiated at the beginning of the diffusion before a constant stimulating polarization (100 mV) was applied at the center of the secondary particles (at bottom‐left) to drive Li^+^ diffusion toward the particle surface. The diffusion across the grain boundary was prohibited [38]. The fastest anisotropic diffusion direction has been set to be along the longest direction and the slowest was set to be perpendicular to the longest direction of any primary particles to simulate anisotropic direction of Li^+^ diffusion inside a particle [59].

## Conflicts of Interest

The authors declare no conflicts of interest.

## Supporting information




**Supporting File**: advs74421‐sup‐0001‐SuppMat.docx.

## Data Availability

The data that support the findings of this study are available from the corresponding author upon reasonable request.
