# Supplementary material for: Boosting Fast‐Charging Performance of Ni‐Rich NCM9055 Cathodes with Nb2O5 Dual Functional Modification
Source: Adv Sci (Weinh). 2026 Mar 12;13(23):e22771. doi: 10.1002/advs.202522771 (PMC13104111; doi:10.1002/advs.202522771)
Supplement: Supplementary file 1 — Supporting File: advs74421‐sup‐0001‐SuppMat.docx. [file ADVS-13-e22771-s001.docx]

**Boosting Fast-Charging Performance of Ni-Rich NCM9055 Cathodes with Nb₂O₅ Dual Functional Modification**

Tian Rao ^1,2,3^, Zhaowen Bai ^4,5^, Jian Wang ^2^, Yang Ren ^5^, Qingsong Weng ^6^, Zhongzhu Liu ^7^, Maxim Avdeev ^8^, Robson Monteiro ^9^, Luanna Parreira ^10^, Xuejie Huang ^3,^*, Guohua Chen ^2,11,^*, Yongming Zhu ^1,^*

Affiliations:

1. School of Chemical Engineering and Chemistry, Harbin Institute of Technology. Harbin, Heilongjiang, China.

2. School of Energy and Environment, City University of Hong Kong, Kowloon, Hong Kong SAR, China.

3. Songshan Lake Material Laboratory, Dongguan, Guangdong, China.

4. Institute of High Energy Physics, Chinese Academy of Sciences, 19B Yuquan Road, Beijing, China.

5. Department of Physics, JC STEM Lab of Energy and Materials Physics, City University of Hong Kong, Hong Kong, China.

6. Department of Mechanical Engineering, Research Institute for Smart Energy (RISE), The Hong Kong Polytechnic University, Kowloon, Hong Kong SAR, China.

7. CITIC Metal, Room 1901, Capital Mansion, Chaoyang, Beijing, China.

8. Australian Nuclear Science and Technology Organization (ANSTO), New Illawarra Road, Lucas Heights, New South Wales, Australia.

9. CBMM North America Inc., Houston, Texas, United States of America.

10. Companhia Brasileira de Mineração e Metalurgia, Araxã, Brazil.

11. Department of Chemical and Biological Engineering, The Hong Kong University of Science and Technology, Clear Water Bay, Kowloon, Hong Kong SAR, China.

(Authors marked with * are corresponding authors.)


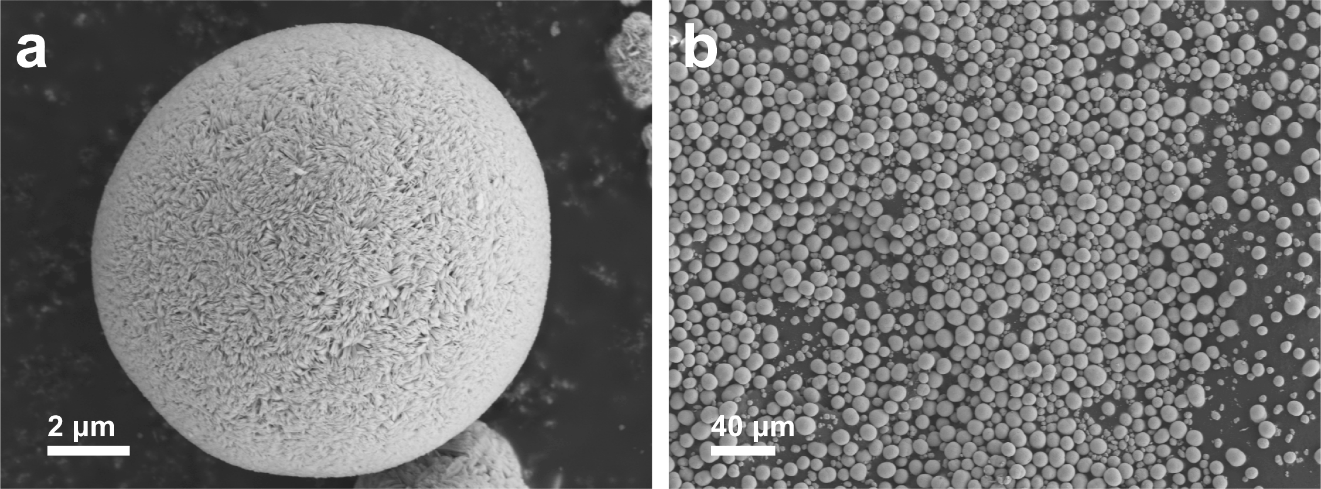


Figure S1.
SEM image showing (a) morphology of synthesized precursor particle with radial structure and (b) showing even secondary particle distribution.


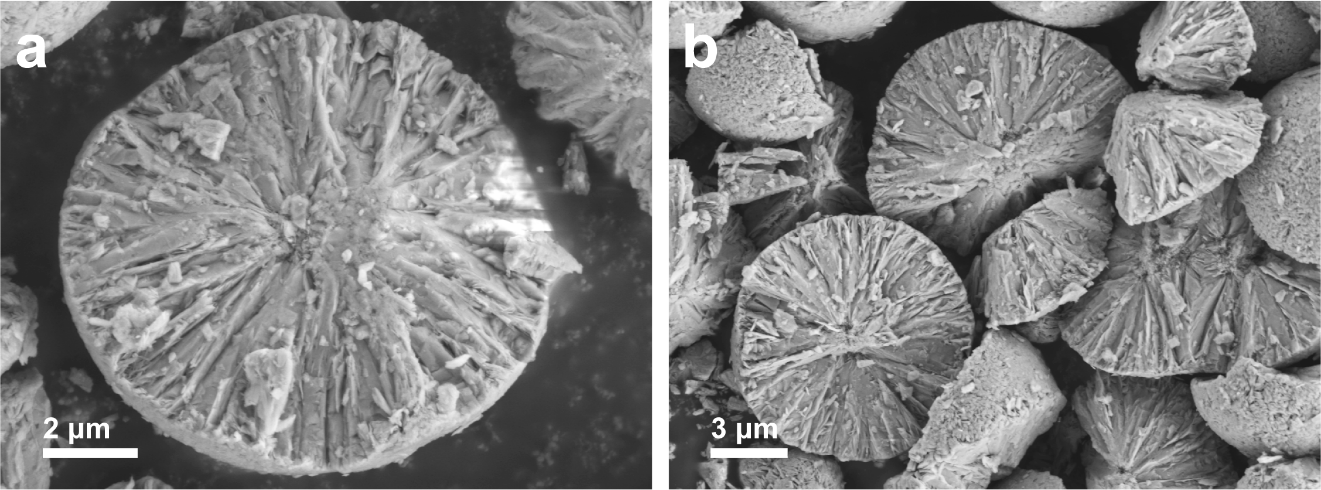


Figure S2.
SEM image of (a) cross-section of synthesized precursor showing radial structure and (b) a lower magnification view. The cross-section sample of precursor was prepared by proper mechanical grinding and cracking of the precursor powder shown in Figure S1.


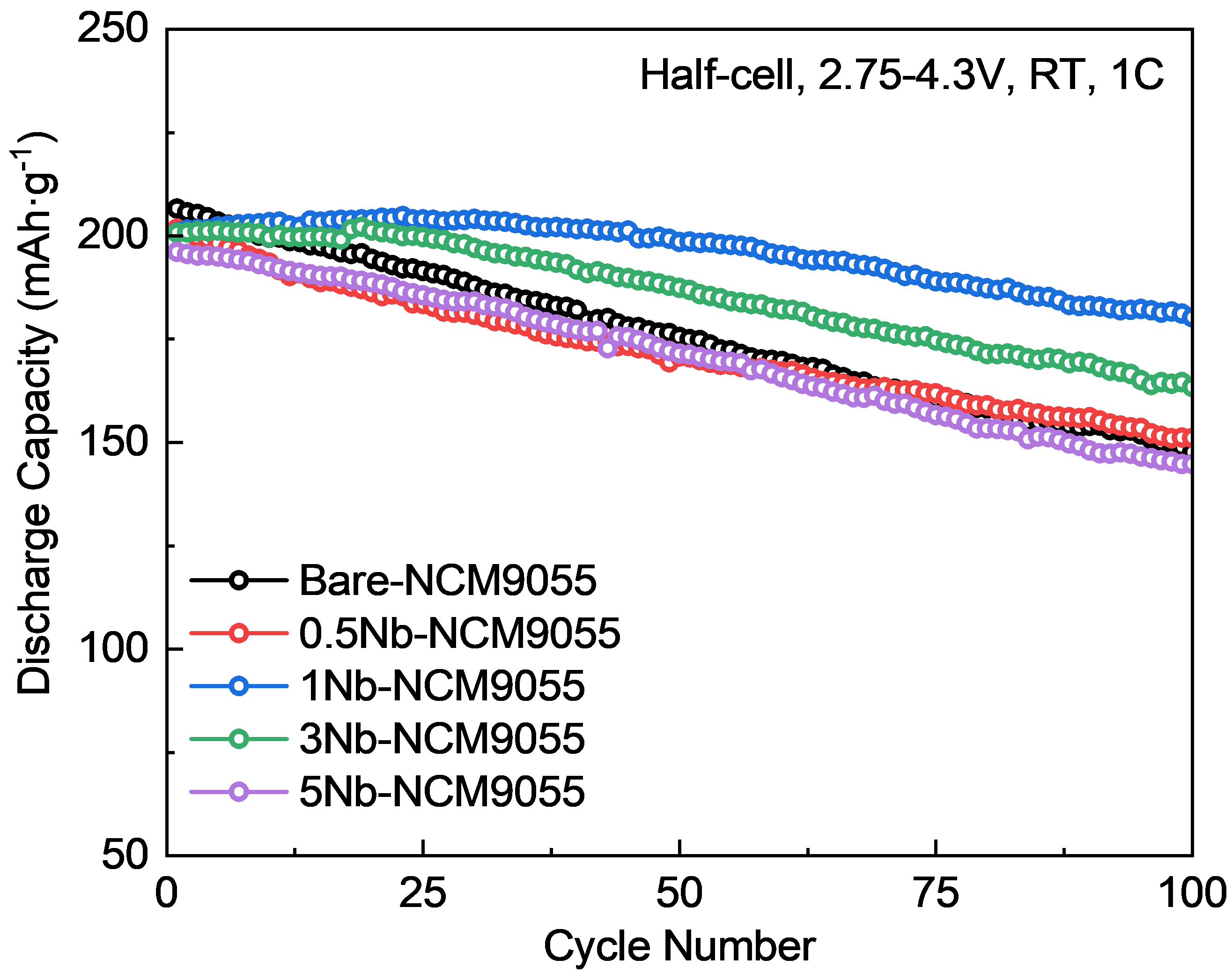


Figure S3.
Cycling capacity retention curve of no modification (marked as “Bare-NCM9055” in figure legend) and 0.5, 1, 3 and 5 mol% Nb modified (marked as “0.5Nb-NCM9055, 1Nb-NCM9055, 3Nb-NCM9055, and 5Nb-NCM9055” in figure legend) NCM9055 samples in half-cells.


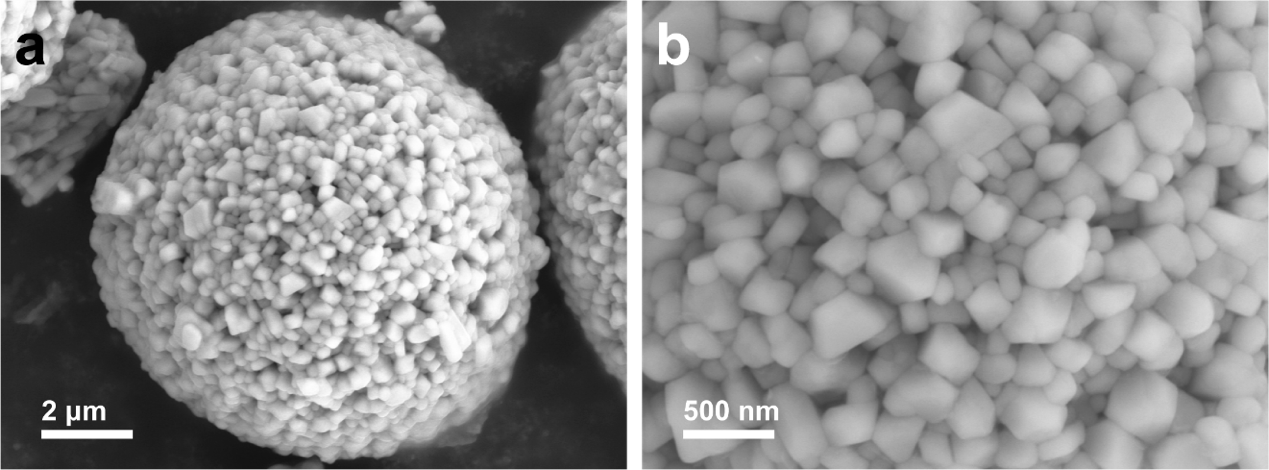


Figure S4.
SEM image of (a) Bare-NCM9055 particle and (b) magnified view of primary particles on Bare-NCM9055.


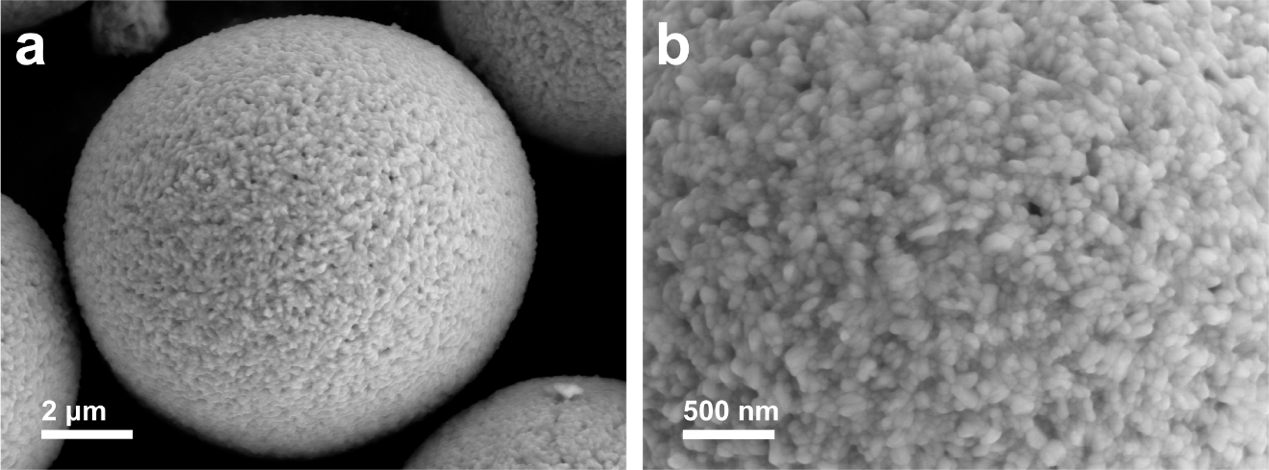


Figure S5.
SEM image of (a) Nb-NCM9055 particle and (b) magnified view of primary particles on Nb-NCM9055.


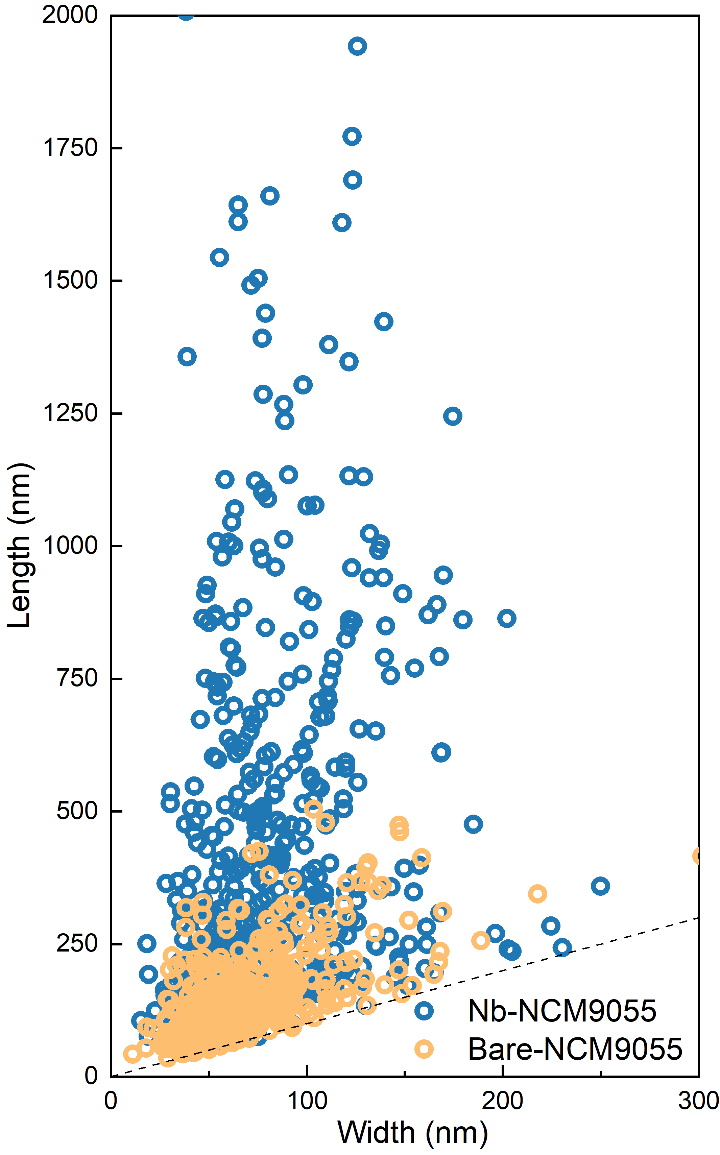


Figure S6.
Quantification results of the corresponding particle length and width from Figure 1a_1_ and 1b_1_.


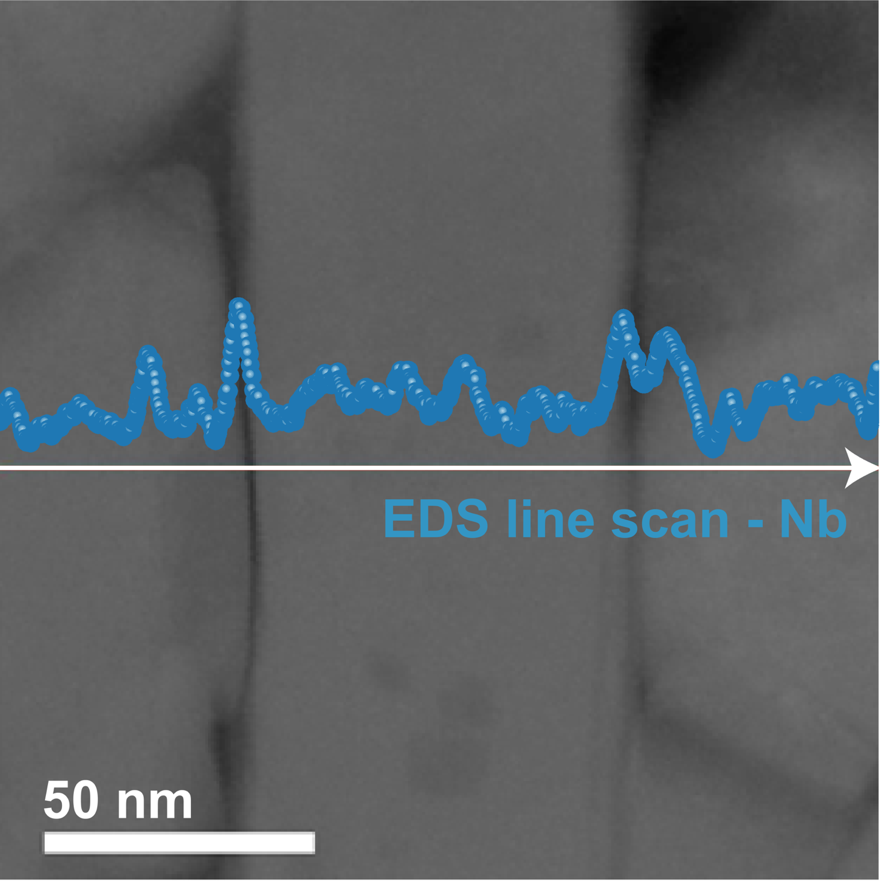


Figure S7.
STEM EDS line scan of grain boundary region for Nb-NCM9055 cathode.


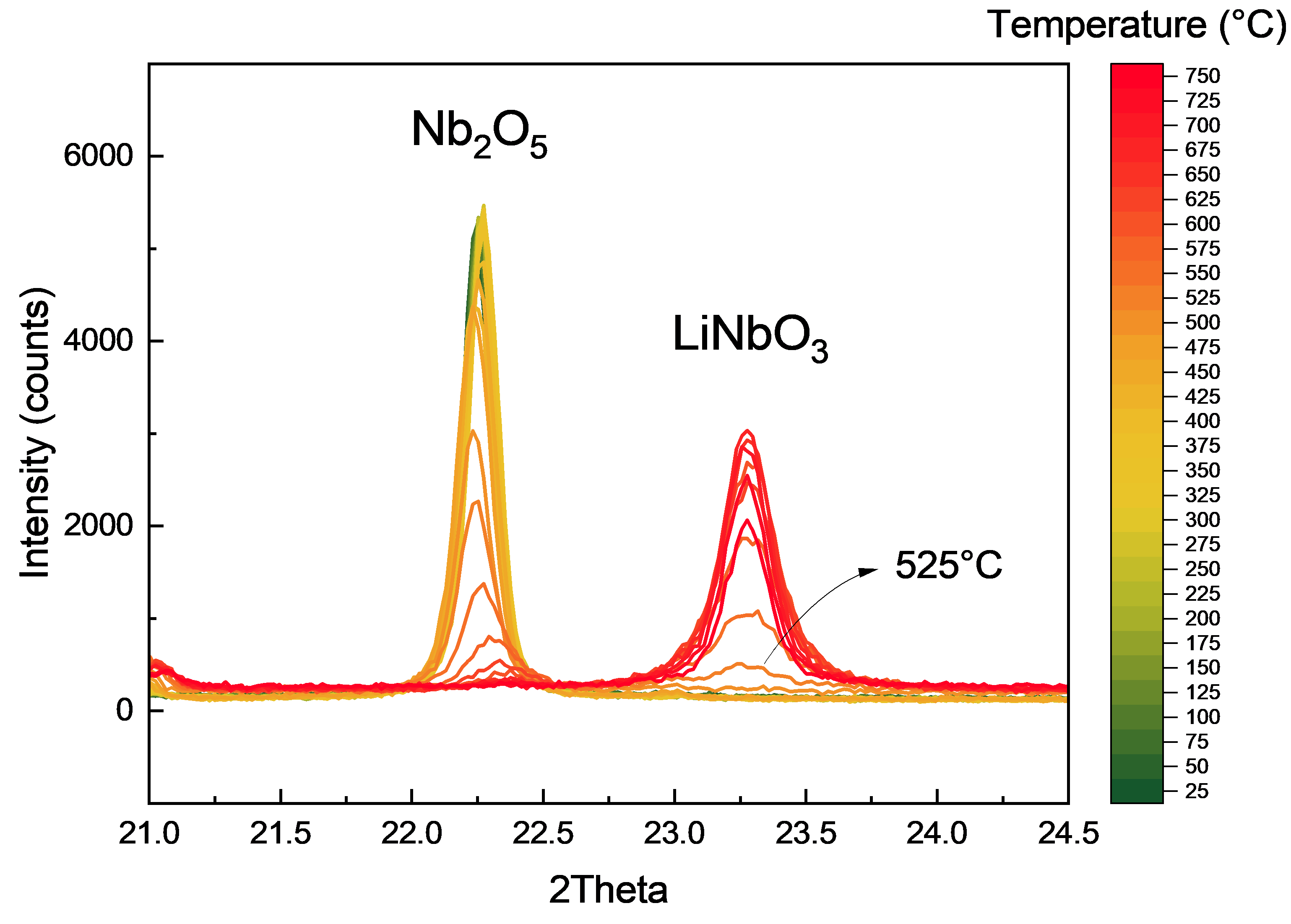


Figure S8.
Enlarged view of in-situ heating XRD pattern of Nb_2_O_5_ in $LiOH\cdot H_{2}O$ from 25 °C to 750 °C between 2-Theta of 21.0 and 24.5 degree.


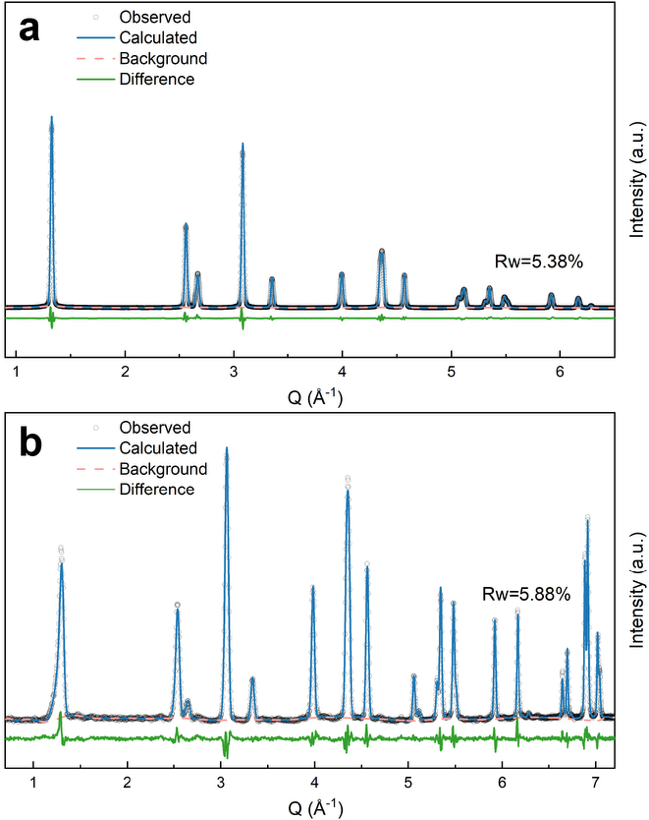


Figure S9.
(a) Synchrotron XRD (SXRD) and (b) powder neutron diffraction (PND) pattern of Bare-NCM9055 sample.


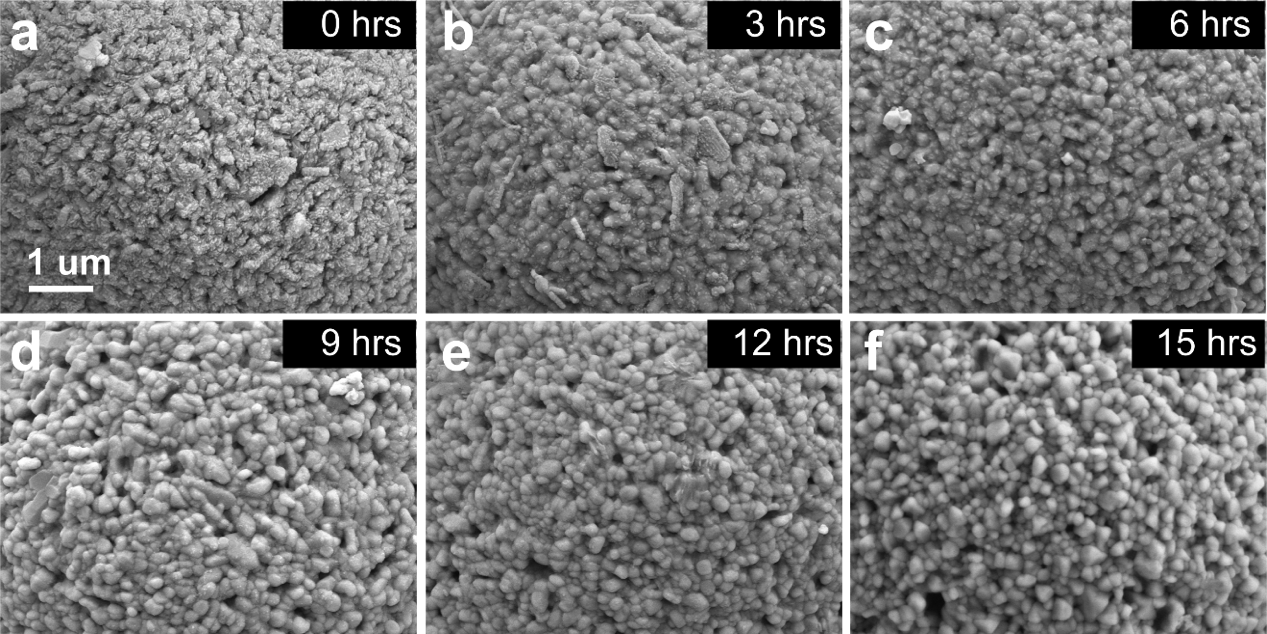


Figure S10.
(a-f) SEM image of Nb-NCM9055 sample calcined at 725 °C for 0, 3, 6, 12, 15 hours, respectively. The “0, 3, 6, 12, 15 hrs” labels denote the onset of the 725 °C calcination, after the lower-temperature pre-calcination step has been completed. All the images in Figure S10 (a-f) share the same scale bar.


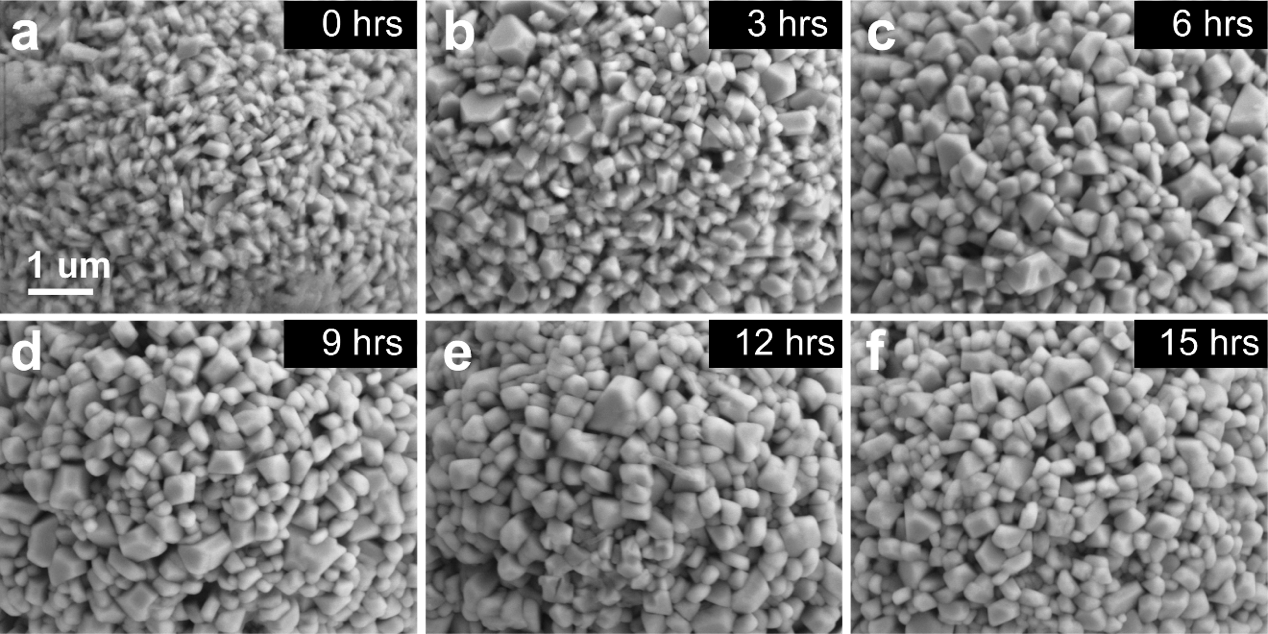


Figure S11.
(a-f) SEM image of Bare-NCM9055 sample calcined at 725 °C for 0, 3, 6, 12, 15 hours, respectively. The “0, 3, 6, 12, 15 hrs” labels denote the onset of the 725 °C calcination, after the lower-temperature pre-calcination step has been completed. All the images in Figure S11 (a-f) share the same scale bar.


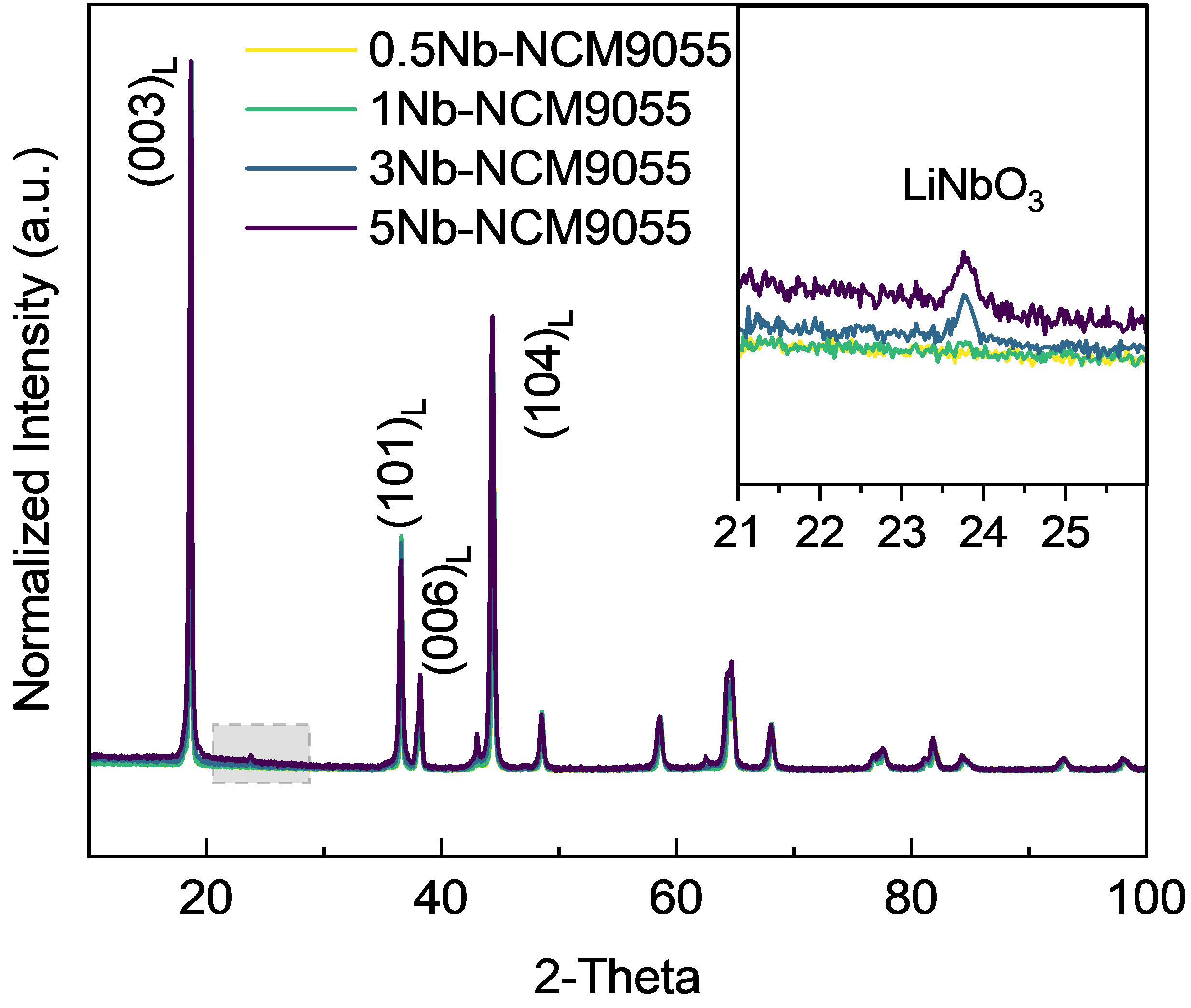


Figure S12.
XRD pattern of synthesized NCM9055 with 0.5, 1, 3 and 5 mol% Nb doping (marked as “0.5Nb-NCM9055, 1Nb-NCM9055, 3Nb-NCM9055, and 5Nb-NCM9055” in figure legend) The inset was a magnified view of LiNbO_3_ peak region. The intensities were normalized using (003) peak.


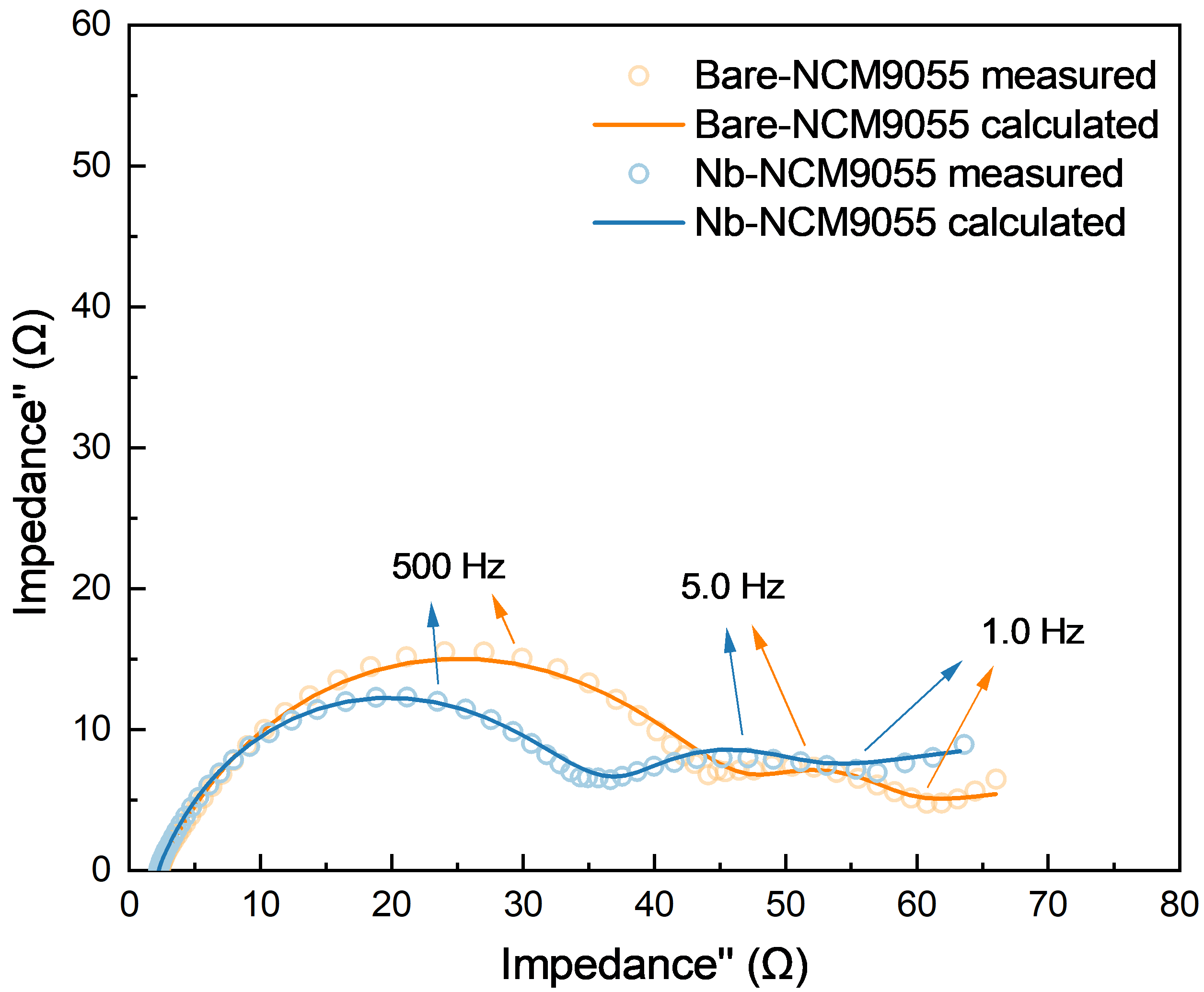


Figure S13.
EIS Nyquist plot and fitted curve for Bare-NCM9055 and Nb-NCM9055 samples at 4.0 V.


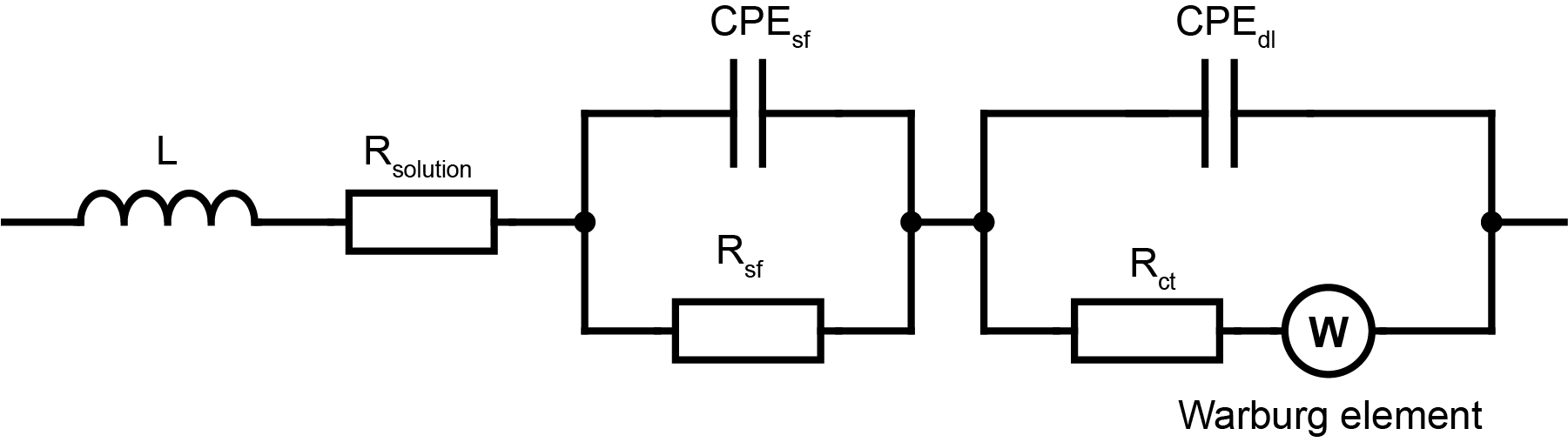


Figure S14.
Equivalent circuit used for EIS fitting. R_solution_, R_sf_ and R_ct_ represent the resistance of solution, surface film and charge transfer process, respectively. CPE_sf_ and CPE_dl_ are constant phase elements which represent the capacitance of surface film and double layer, respectively.


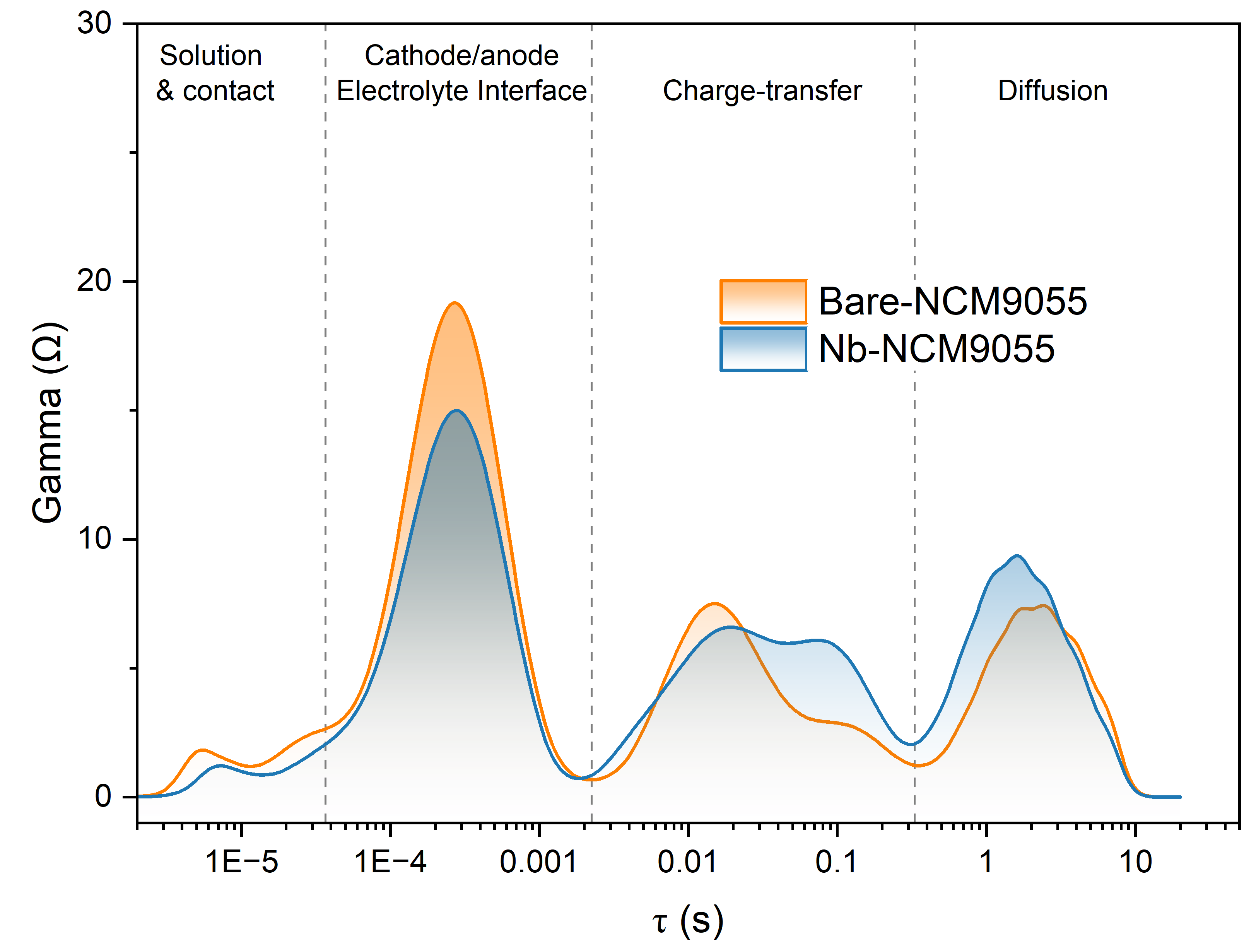


Figure S15.
Distribution of Relaxation Times (DRT) analysis from the electrochemical impedance spectroscopy (EIS) data of Bare-NCM9055 and Nb-NCM9055 samples at 4.0 V


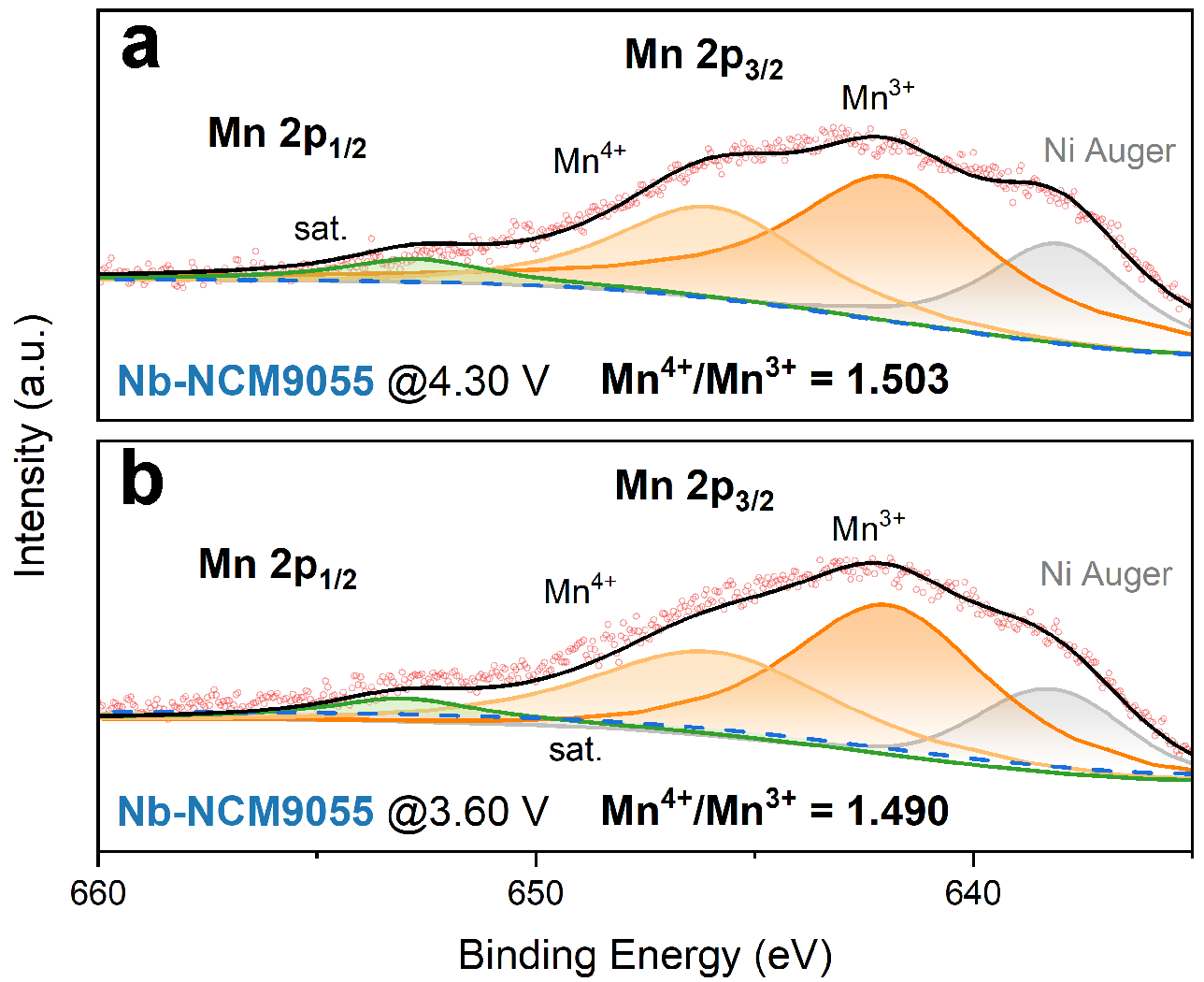


Figure S16.
XPS Mn 2p spectrum and fitting result of Nb-NCM9055 electrode (a) 4.30 V and (b) 3.60 V. Etching time = 200 s for both samples. Note that the peak appearing at ~ 648 eV (highlighted in grey) originated from the Ni LMM Auger.


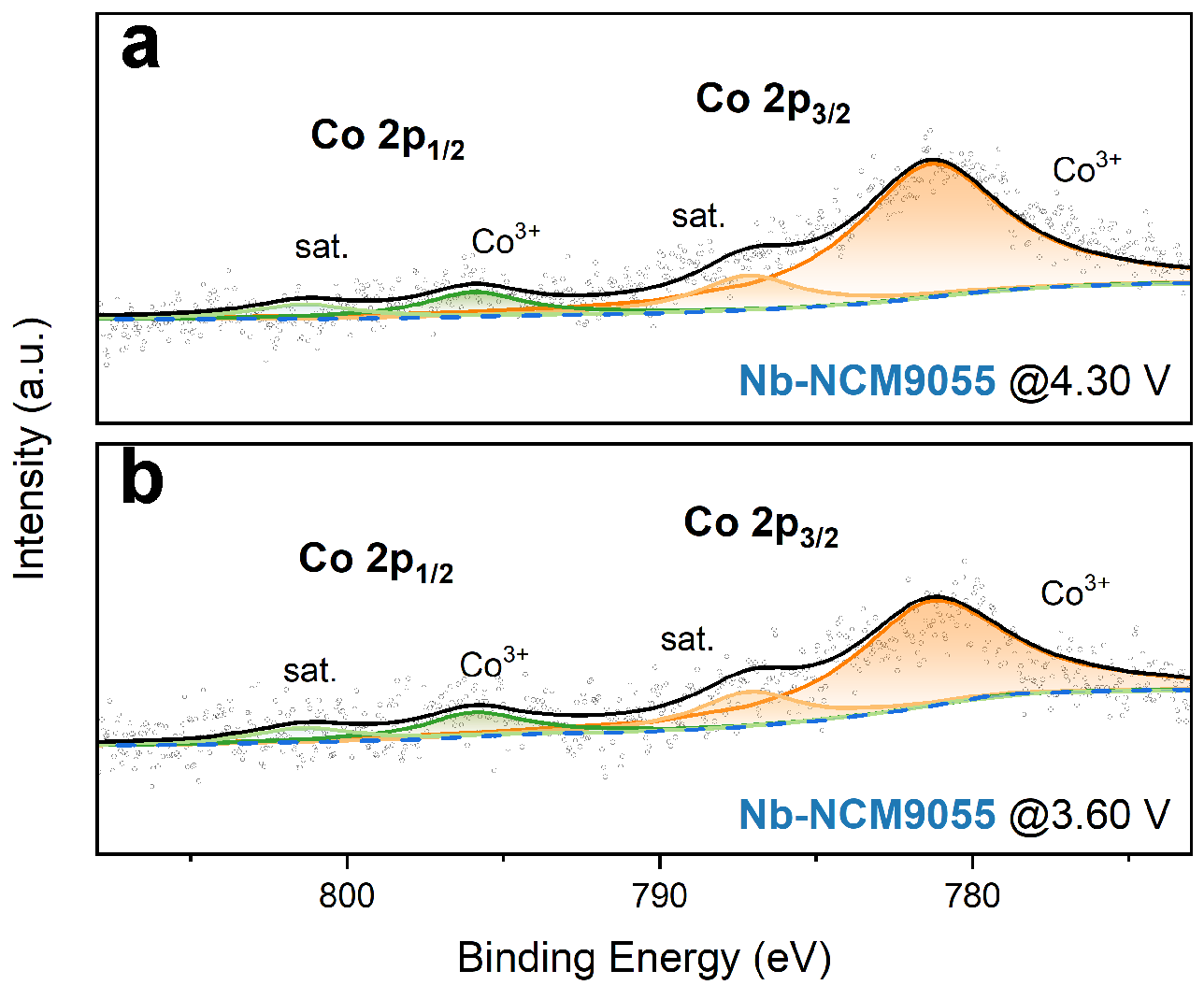


Figure S17.
XPS Co 2p spectrum and fitting result of Nb-NCM9055 electrode at (a) 4.30 V and (b) 3.60 V. Etching time = 200 s for both samples.


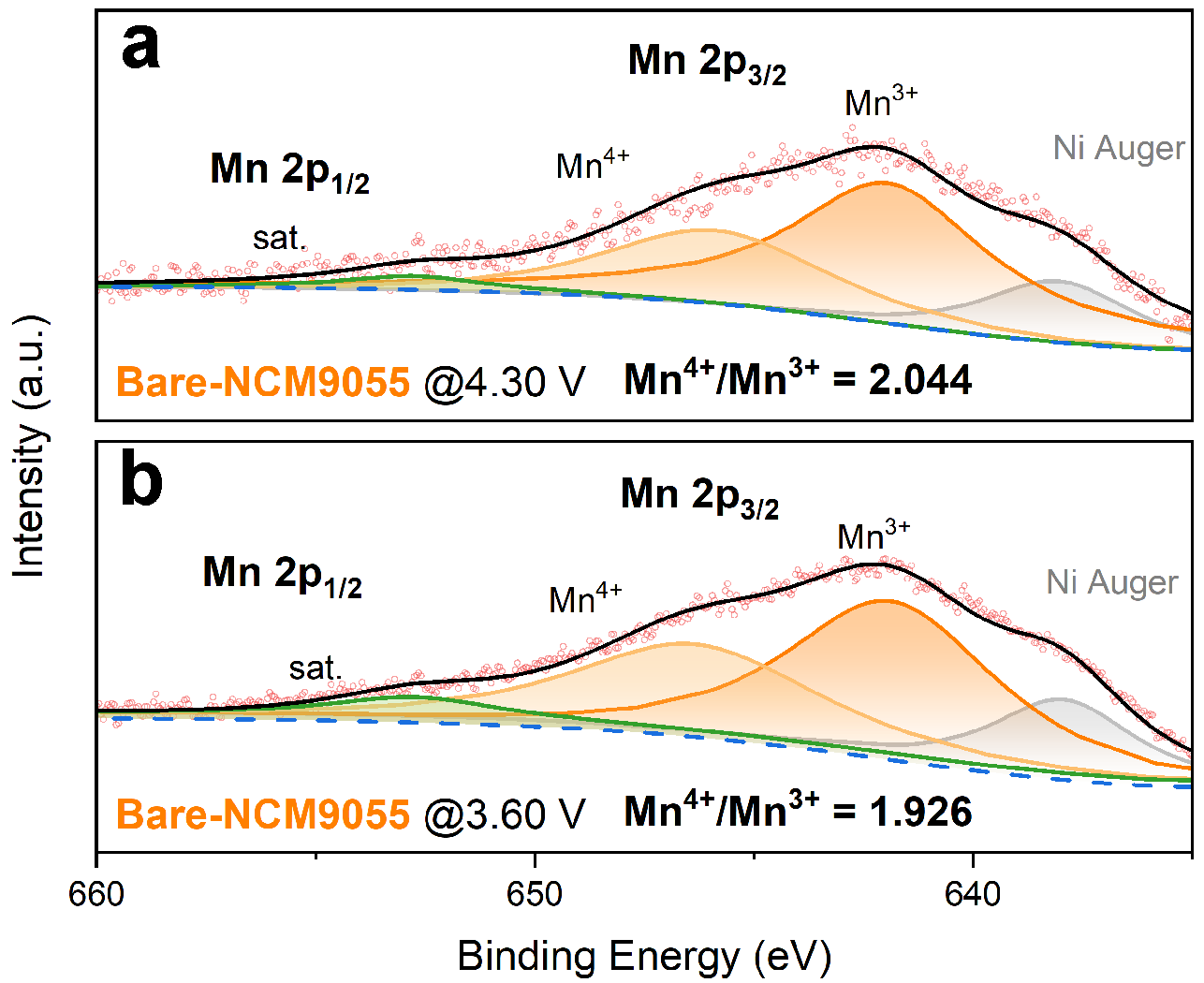


Figure S18.
XPS Mn 2p spectrum and fitting result of Bare-NCM9055 electrode at (a) 4.30 V and (b) 3.60 V. Etching time = 200 s for both samples. Note that the peak appearing at ~ 648 eV (highlighted in grey) originated from the Ni LMM Auger.


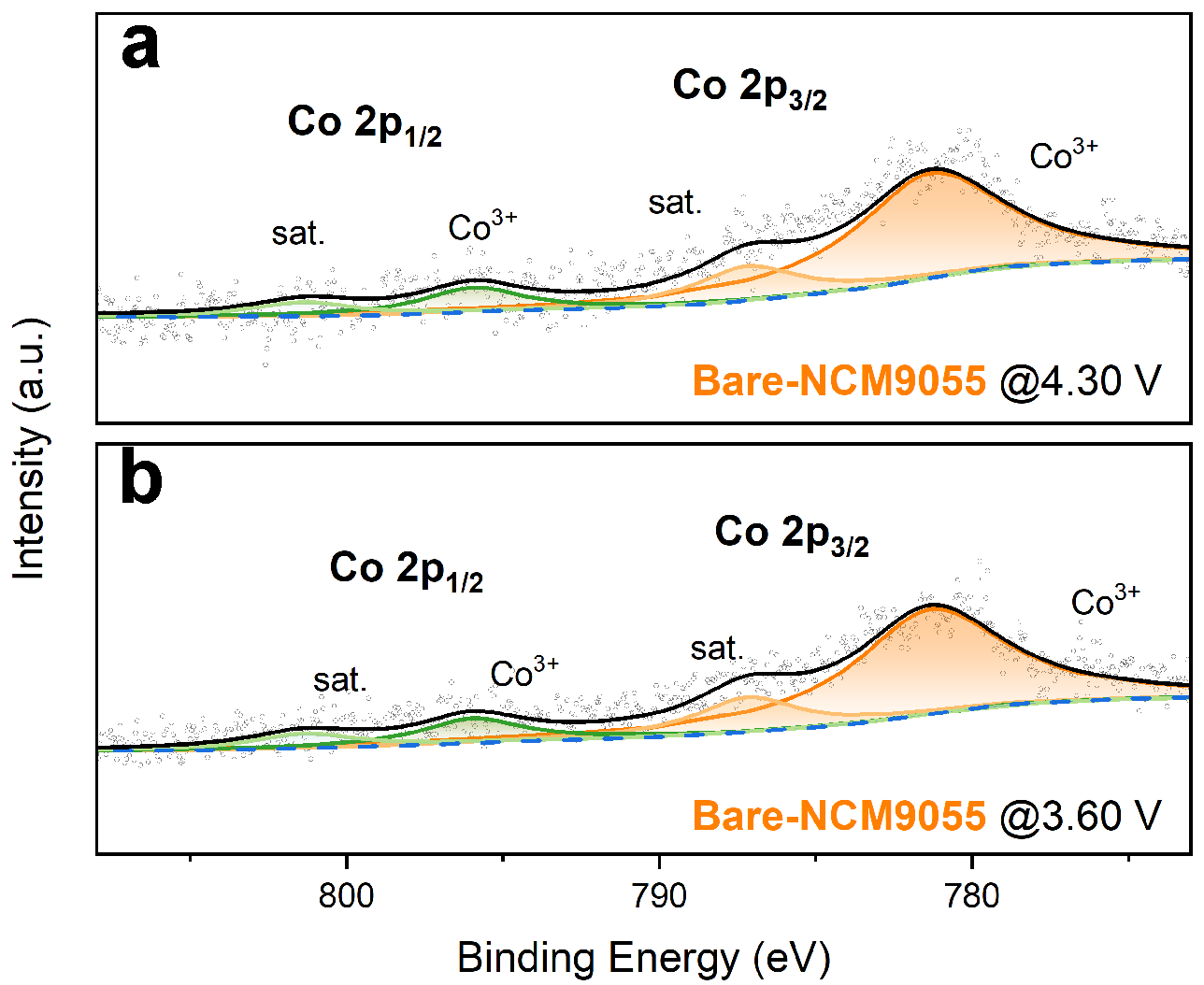


Figure S19.
XPS Co 2p spectrum and fitting result of Bare-NCM9055 electrode at (a) 4.30 V and (b) 3.60 V. Etching time = 200 s for both samples.


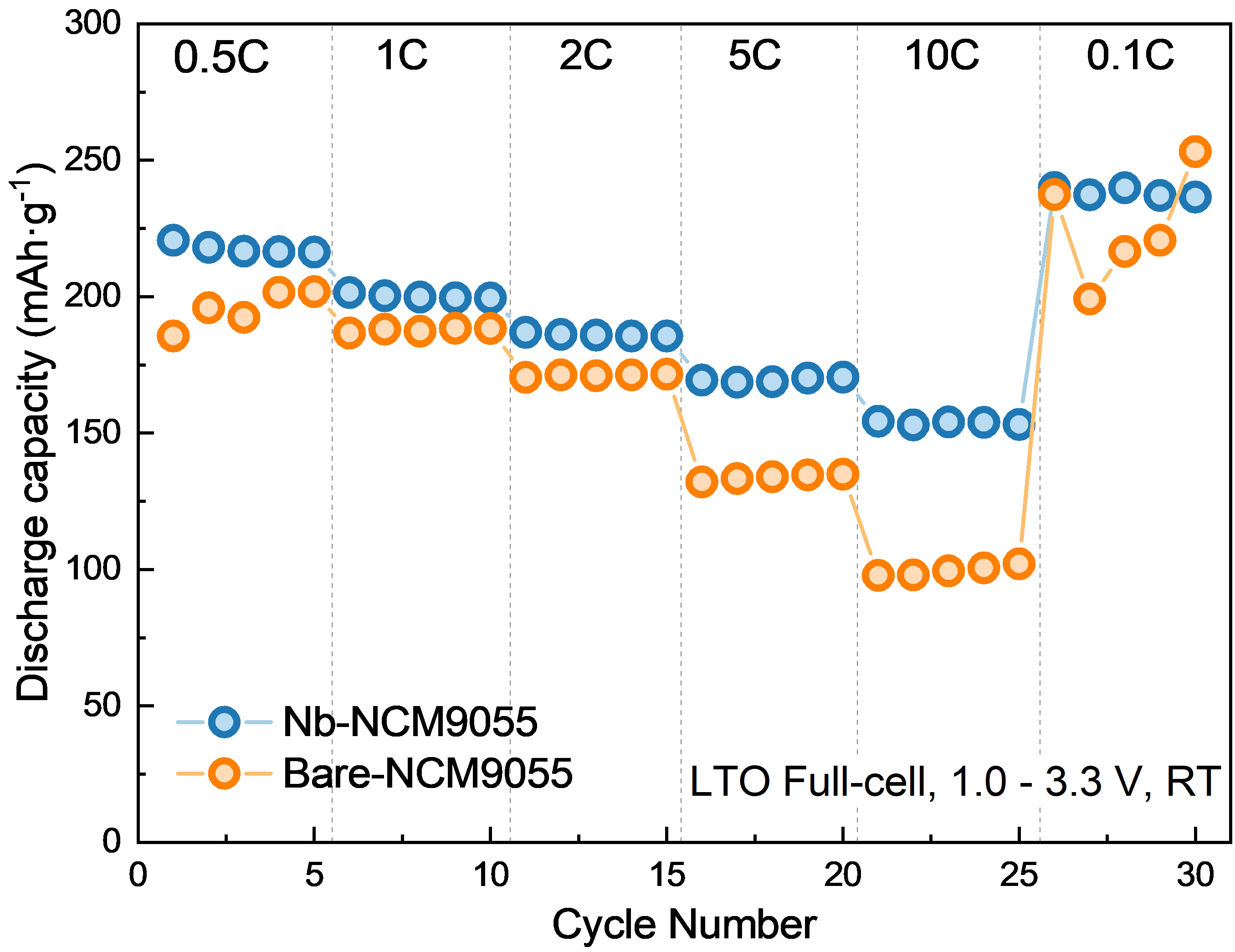


Figure S20.
Reversible discharge capacity of Nb-NCM9055 and Bare-NCM9055 LTO | NCM9055 full-cells at different rates.


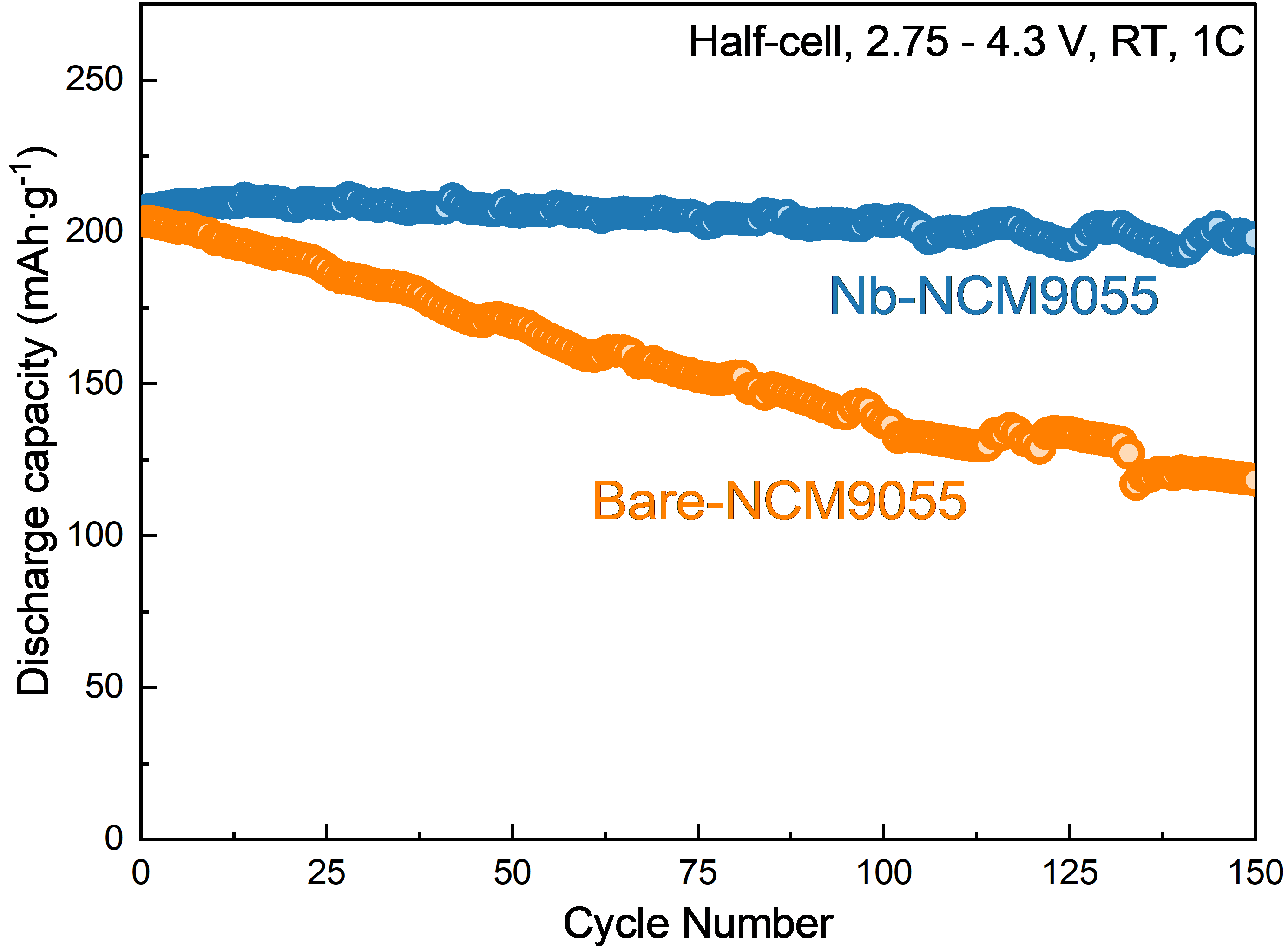


Figure S21.
Cycling capacity retention curve of Nb-NCM9055 and Bare-NCM9055 half-cells at 1 C.


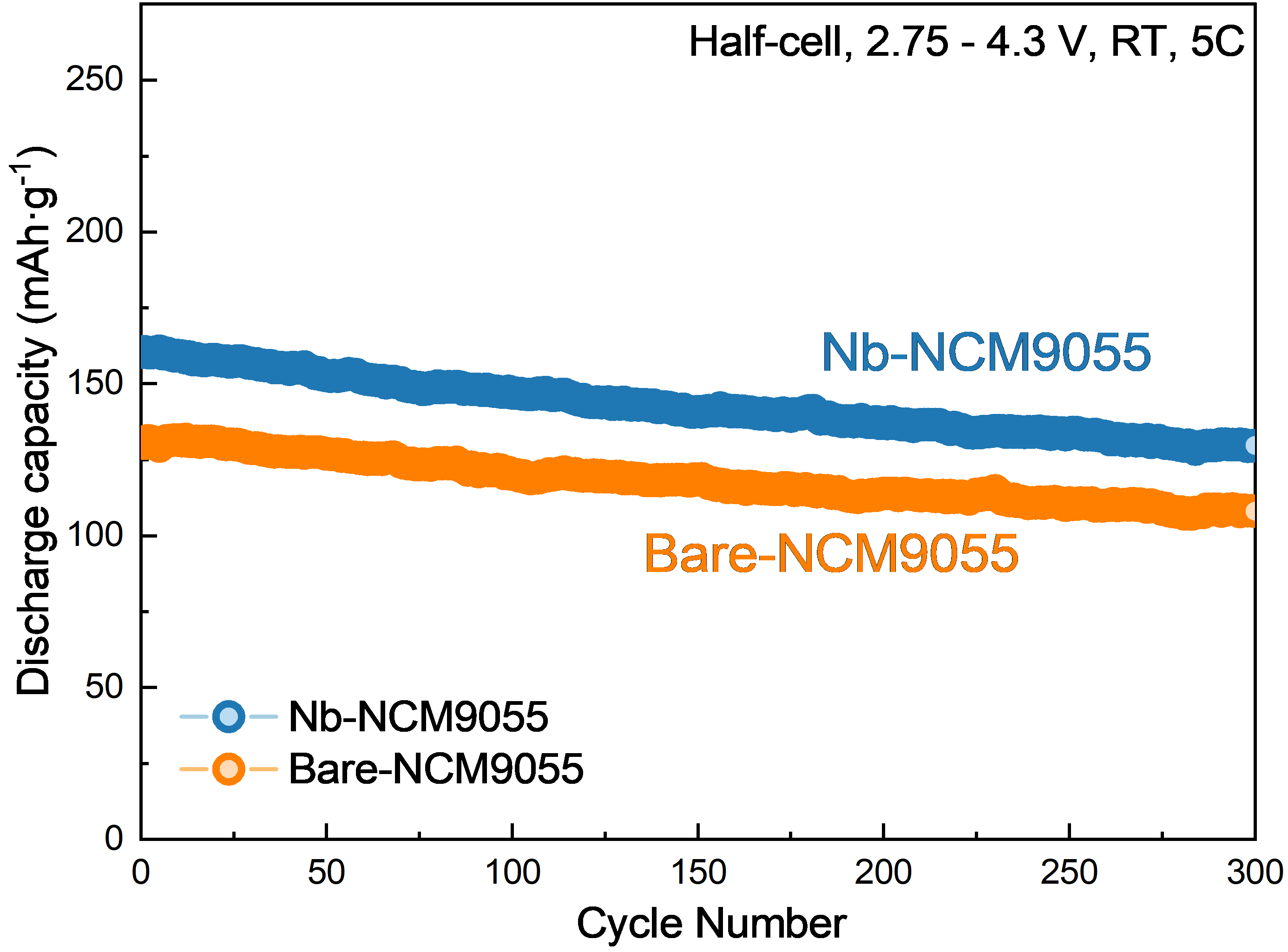


Figure S22.
Cycling capacity retention curve of Nb-NCM9055 and Bare-NCM9055 half-cells at 5 C.


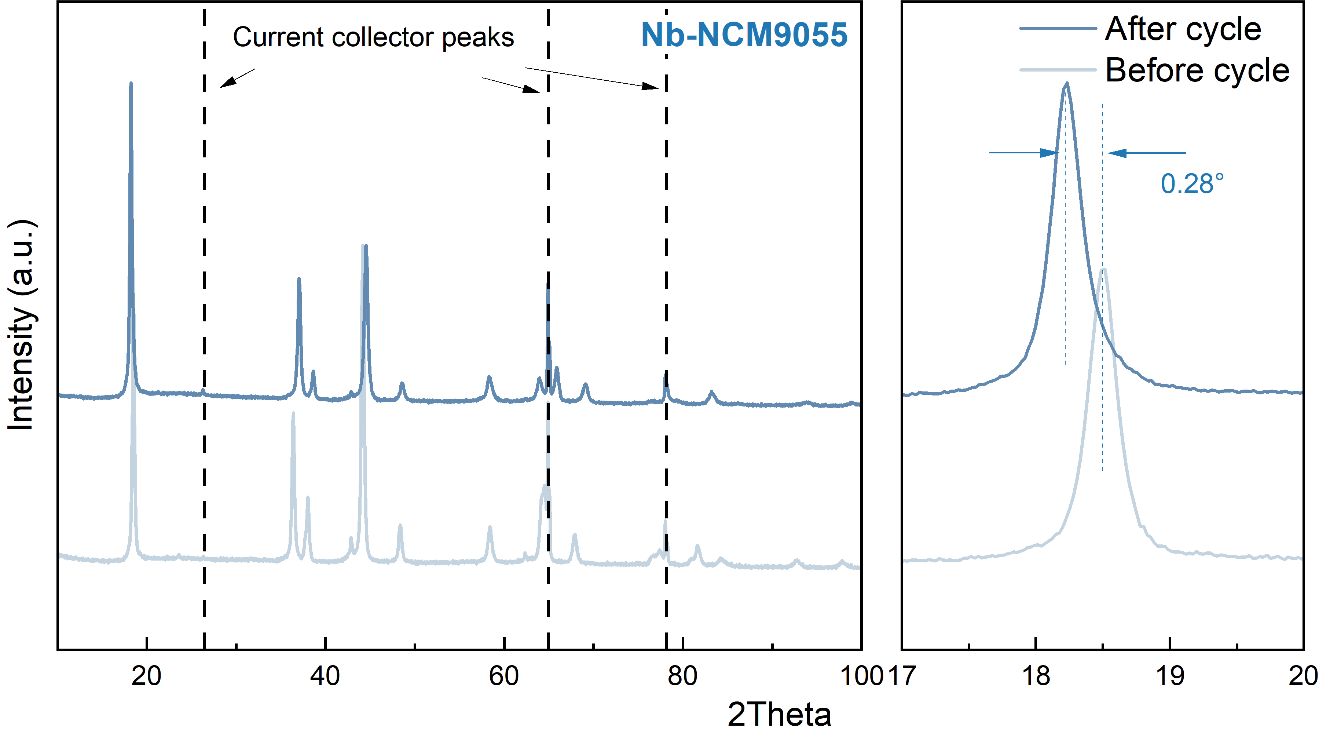


Figure S23.
XRD pattern of Nb-NCM9055 before and after 500 cycles at 5 C.


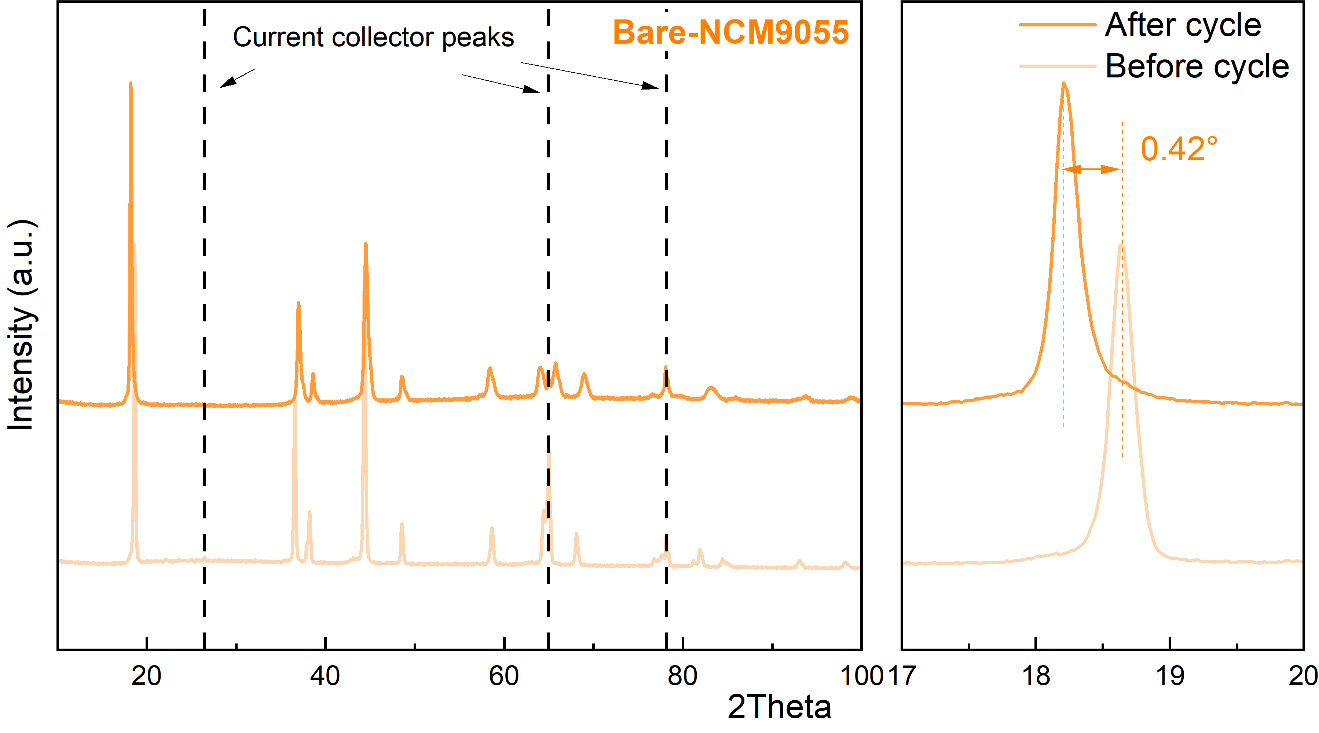


Figure S24.
XRD pattern of Bare-NCM9055 before and after 500 cycles at 5 C.


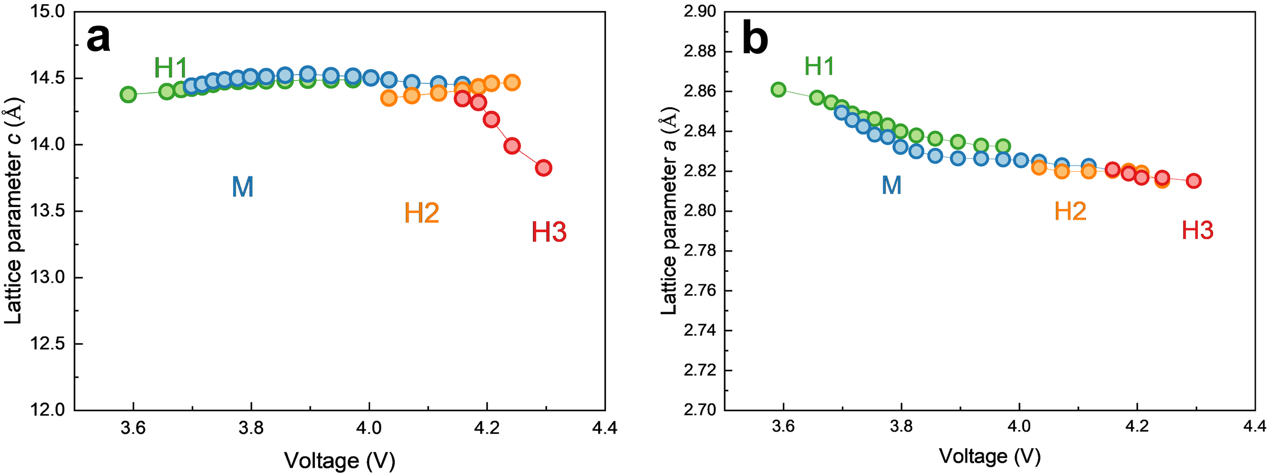


Figure S25.
The Rietveld refinement result of Nb-NCM9055 sample while charging. (a) lattice parameter *c*, (b) lattice parameter *a*.


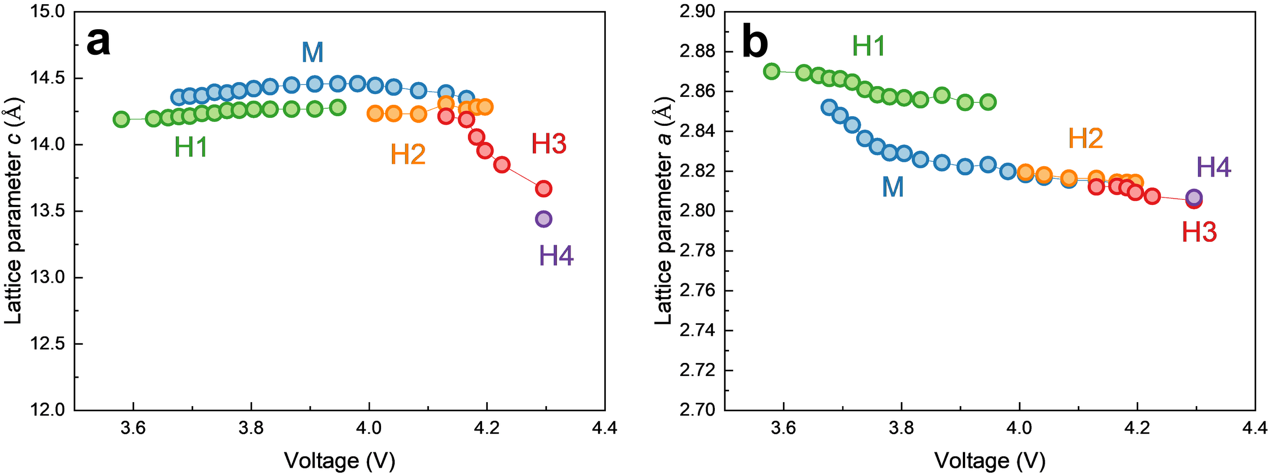


Figure S26.
The Rietveld refinement result of Bare-NCM9055 sample while charging. (a) lattice parameter *c*, (b) lattice parameter *a*.


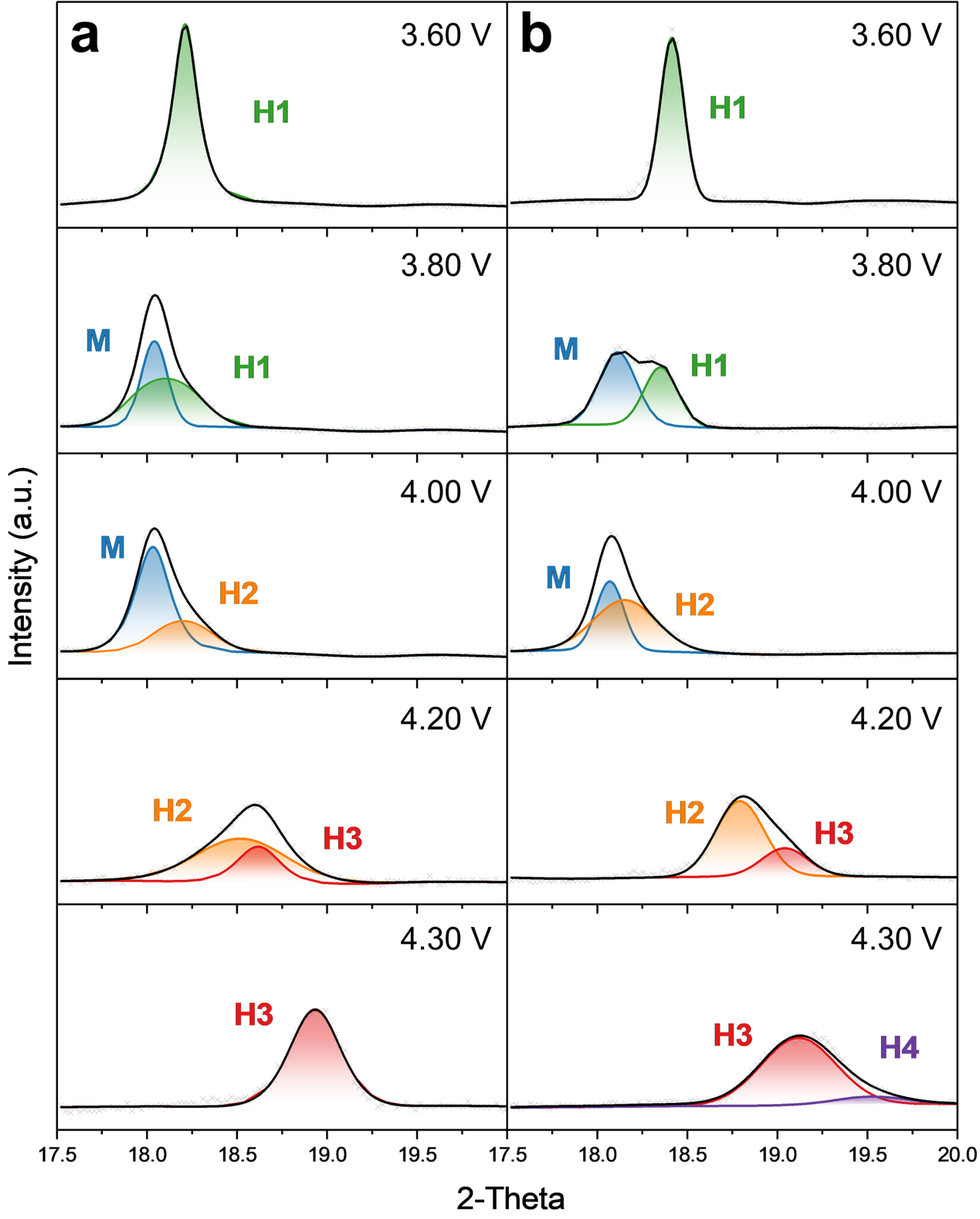


Figure S27.
Enlarged view of (003) peak in-situ XRD data of (a) Nb-NCM9055 and (b) Bare-NCM9055 sample at different voltage.


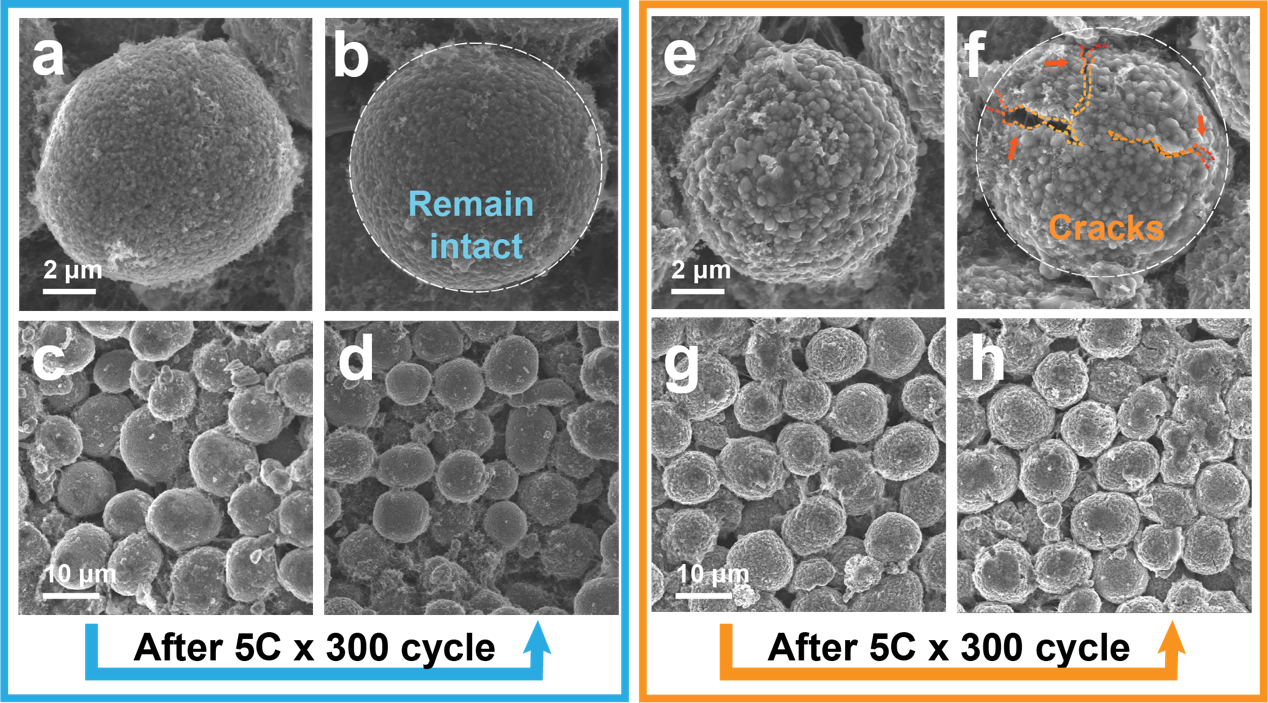


Figure S28.
SEM top-view for electrode before and after 300 cycles (a-d) Nb-NCM9055, (e-h) Bare-NCM9055

Table S1. ICP-AES result of Bare-NCM9055 and Nb-NCM9055 sample. Transition metal means the combined concentration of Ni, Co, Mn and Nb element.

| Sample | Ni / Transition metal (%) | Co / Transition metal (%) | Mn / Transition metal (%) | Nb / Transition metal (%) |
| --- | --- | --- | --- | --- |
| Nb-NCM9055 | 89.409 | 4.830 | 4.811 | 0.950 |
| Bare-NCM9055 | 91.741 | 4.117 | 4.142 | N/A |

Table S2. Combined Rietveld refinement result of Nb-NCM9055 SXRD and PND pattern.

| Source | Lattice parameter (Å) | | Volume (Å^3^) | Nb occupancy in TM site (atom %) | Li/Ni antisite (atom%) | Phase fraction | |
| --- | --- | --- | --- | --- | --- | --- | --- |
|  | a | c |  |  |  | Nb-NCM9055 (R-3m) | LiNbO_3_ (R3c) |
| SXRD & PND | 2.87723(8) | 14.2018(3) | 101.818(3) | 0.89 | 4.50 | 0.9989 | 0.0011 |

R_w_ for SXRD and NPD refinement: 6.96 % and 7.92 %. Total R_w_: 7.84%.

Table S3. Combined Rietveld refinement result of Bare-NCM9055 SXRD and PND pattern

| Source | Lattice parameter (Å) | | Volume (Å^3^) | Nb occupancy in TM site (atom %) | Li/Ni antisite (atom%) | Phase fraction | |
| --- | --- | --- | --- | --- | --- | --- | --- |
|  | a | c |  |  |  | Nb-NCM9055 (R-3m) | LiNbO_3_ (R3c) |
| SXRD & PND | 2.87409(2) | 14.1942(1) | 101.542(1) | N/A | 1.53 | 1 | N/A |

R_w_ for SXRD and NPD refinement: 5.38 % and 5.88 %. Total R_w_: 5.49%.

Table S4. EIS fitting result of Bare-NCM9055 and Nb-NCM9055 sample.

|  | Nb-NCM9055 | Bare-NCM9055 |
| --- | --- | --- |
| R_solution_ (Ω) | 1.985 | 1.888 |
| L (H) | 2.42E-07 | 2.68E-07 |
| CPE_sf_, *Y_0_* (S s*^n^*) | 0.01887 | 0.04135 |
| CPE_sf_, *n* | 0.3825 | 0.2822 |
| R_SEI_ (Ω) | 48.97 | 58.64 |
| CPE_dl_, *Y_0_* (S s*^n^*) | 3.96E-05 | 4.32E-05 |
| CPE_dl_, *n* | 0.8058 | 0.7626 |
| R_ct_ (Ω) | 29.72 | 40.75 |
| W_O_, *Y_0_* (S s^5^) | 0.03942 | 0.03484 |
| W_O_, *B* (s^5^) | 0.2484 | 0.2413 |

Table S5. Performance data from published papers about Nb modification

| Base material | Cycle performance | | | | Rate performance | | Source |
| --- | --- | --- | --- | --- | --- | --- | --- |
|  | Initial capacity (mAh g^-1^) | Temperature | C-rate | Capacity retention | C-rate | Capacity (mAh g^-1^) |  |
| LiNi_0.94_Co_0.02_Al_0.04_O_2_ (NCA94) | 193.9 | RT | 2 C | 88.7 % after 300 cycles | 5 C | 186 | ^[1]^ |
| LiNi_0.83_Co_0.11_Mn_0.06_O_2_ (NCM) | 190 | 25 °C | 1.0 C | 86.6 % after 200 cycles | 5.0 C | 154.6 | ^[2]^ |
| LiNi_0.8_Co_0.1_Mn_0.1_O_2_ (NCM811) | 164.9 | RT | 1 C | 96.9 % after 300 cycles | 2 C | 150.3 | ^[3]^ |
| LiNi_0.8_Co_0.1_Mn_0.1_O_2_ (NCM) | 202.3 | RT | 0.1 C | 90.6 % after 100 cycles | 5.0 C | ~170 | ^[4]^ |
| LiNi_0.85_Co_0.1_Al_0.05_O_2_ (N85CA) | 192 | RT | 0.5 C | 94.19 % after 100 cycles | 5 C | 156.2 | ^[5]^ |
| LiNi_0.8_Co_0.1_Mn_0.1_O_2_ (NCM811) | 181.6 | RT | 1 C | 94.55 % after 100 cycles | 5 C | 151.46 | ^[6]^ |
| LiNi_0.6_Co_0.2_Mn_0.2_O_2_ (NCM622) | ~145 | 60 °C | 2 C | 91.4 % after 500 cycles |  |  | ^[7]^ |
| LiNi_0.8_Co_0.1_Mn_0.1_O_2_ (NCM811, SC) | ~197 | 25 °C | 1 C | 92.54 % after 100 cycles | 5 C | 162.2 | ^[8]^ |
| LiNi_0.9_Co_0.1_O_2_ (NC, SC) | ~141 | RT | 1 C | 91.9 % after 500 cycles | 5 C | 169.1 | ^[9]^ |
| LiNi_0.8_Co_0.1_Mn_0.1_O_2_ (NCM811) | 233.8 (at 0.1C) | RT |  |  | 10.0 C | 70.2 | ^[10]^ |
| LiNi_0.8_Co_0.1_Mn_0.1_O2(NCM811, SC) | ~191 | 30 °C | 1 C | 80.33 % after 100 cycles |  |  | ^[11]^ |
| LiNi_0.8_5Co_0.1_Mn_0.05_O_2_ (NCM85) | ~185 | 30 °C | 1 C (D) | 97 % after 100 cycles | 5 C | 165 | ^[12]^ |
| LiNi_0.8_Co_0.1_Mn_0.1_O_2_ (NCM811) | 182 | RT | 1 C | 74.17 % after 200 cycles | 10 C | ~128 | ^[13]^ |
| LiNi_0.8_Co_0.1_Mn_0.1_O_2_ (NCM811, SC) | ~175 | RT | 1 C | 93.7 % after 200 cycles | 5 C | 155.5 | ^[14]^ |
| LiNi_0.95_Mn_0.05_O_2_ (NM95) | 190 | RT | 1 C | 96.6 % after 100 cycles | 5 C | 174.4 | ^[15]^ |
| LiNi_0.83_Co_0.12_Mn_0.05_O_2_ (NCM) | 198.6 | 25 °C | 1 C | 92.68 % after 150 cycles | 5 C | 164.1 | ^[16]^ |
| LiNi_0.9_Co_0.05_Mn_0.05_O_2_ (NCM9055) | 189.6 | RT | 5C | 83.0 % after 500 cycles | 10 C | 152.4 | This work |

Supplemental Media 1. Li^+^ diffusion simulation of Nb-NCM9055 with internal radial structure


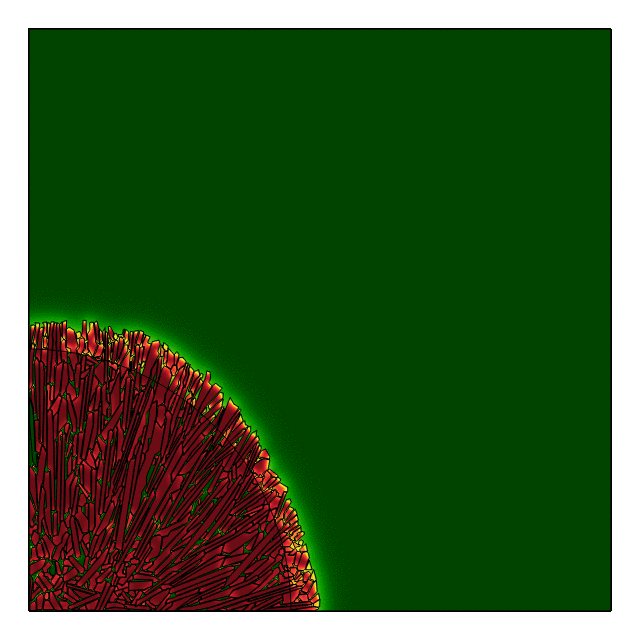


Supplemental Media 2. Li^+^ diffusion simulation of Bare-NCM9055 with conventional structure


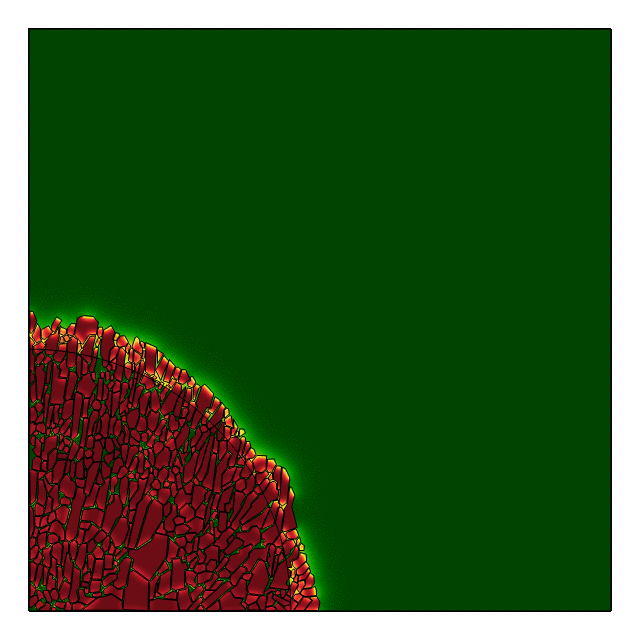


References

[1] X. Li, T. Lai, A. Sheng, J. Yang, Y. Li, S. Xiao, W. Li, H. Tan, B. Huang, *J Energy Storage* **2023**, *72*, 108262.

[2] J. Wang, Z. Yi, C. Liu, M. He, C. Miao, J. Li, G. Xu, W. Xiao, *J Colloid and Interface Sci* **2023**, *635*, 295.

[3] F. Tian, Y. Zhang, Z. Liu, R. de Souza Monteiro, R. M. Ribas, P. Gao, Y. Zhu, H. Yu, L. Ben, X. Huang, *Solid State Ion* **2021**, *359*, 115520.

[4] Y.-R. Kim, Y.-W. Yoo, D.-Y. Hwang, T.-Y. Shim, C.-Y. Kang, H.-J. Park, H.-S. Kim, S.-H. Lee, *Solid State Ion* **2023**, *389*, 116108.

[5] H. He, J. Dong, D. Zhang, C. Chang, *Ceram Int* **2020**, *46*, 24564.

[6] J. Li, M. Zhang, D. Zhang, Y. Yan, Z. Li, *Chem Eng J* **2020**, *402*, 126195.

[7] Sumitomo Metal Mining Co., Ltd., Battery Research Laboratories, 17-3, Isoura-cho, Nihama, Ehime, 792-0008, Japan, H. Kaneda, *Int J Electrochem Sci* **2017**, *12*, 4640.

[8] S. Jamil, M. Fasehullah, B. Jabar, P. Liu, M. K. Aslam, Y. Zhang, S. Bao, M. Xu, *Nano Energy* **2022**, *94*, 106961.

[9] H. Zhu, Z. Wang, L. Chen, Y. Hu, H. Jiang, C. Li, *Adv Mater* **2022**, *35*, 2209357.

[10] B. Wang, K. Li, G. Xu, Z. Zhang, X. Wang, J. Sun, Y. Song, X. Zhang, Y. Liang, D. Kong, Y. Qiu, Q. Teng, X. Cui, J. Chen, J. Zhao, J. Wang, H. Yang, J. Huang, Y. Tang, *Angew Chem Int Ed* **2025**, 64, e202502725.

[11] Z. Wei, Z. Fang, C. Liang, S. Chen, A. Teng, M. Sun, Y. Zhang, Y. Yu, X. Meng, P. Duan, L. Jiang, J. Sun, Q. Wang, *The Innovation Energy* **2025**, 100083.

[12] Y. Levartovsky, A. Chakraborty, S. Kunnikuruvan, S. Maiti, J. Grinblat, M. Talianker, D. T. Major, D. Aurbach, *ACS Appl Mater Interfaces* **2021**, 13, 34145.

[13] Q. Zhang, R. Wang, T. Zhang, Y. Zhang, Y. Lian, W. Zhao, *Ionics* **2020**, *26*, 5971.

[14] C. Sun, W. Chen, P. Gao, H. Hu, J. Zheng, Y. Zhu, *Ionics* **2022**, *28*, 747.

[15] Y. Sun, Y. Wang, S. Wang, X. Li, P. Gao, Y. Zhu, *J Mater Sci* **2023**, *58*, 10428.

[16] H. Wu, X. Zhou, C. Yang, D. Xu, Y.-H. Zhu, T. Zhou, S. Xin, Y. You, *ACS Appl Mater Interfaces* **2023**, *15*, 18828.
